# Impaired nuclear PTEN function drives macrocephaly, lymphadenopathy and late-onset cancer in PTEN hamartoma tumour syndrome

**DOI:** 10.1242/dmm.052527

**Published:** 2026-01-07

**Authors:** Priyanka Tibarewal, Victoria Rathbone, Sarah E. Conduit, Gala Anastasia Electra Classen, Fiona Black, Mohammad Amin Danesh, Georgia Constantinou, Zeinab Asgarian, Koujiro Tohyama, Nisha Kriplani, Virginia Alvarez Garcia, Elizabeth Foxall, Djenat Belarbi, Marie Leverve, Wayne Pearce, Mahreen Adil, Zofia Varyova, Lucia Conde, Adriana Alves, Glenn R. Masson, Roger L. Williams, Adrienne M. Flanagan, Javier Herrero, Isra Ahmed Mohamed, Katerina Stroud, Marc Tischkowitz, Katherine Lachlan, Cheryl L. Scudamore, Mark G. H. Scott, Nicholas R. Leslie, Nicoletta Kessaris, Bart Vanhaesebroeck

**Affiliations:** ^1^ Cancer Institute, University College London, London WC1E 6BT, UK; ^2^ Wolfson Institute of Biomedical Research, University College London, London WC1E 6BT, UK; ^3^ Department of Physiology, School of Dentistry, Iwate Medical University, Yahaba 028-3694, Japan; ^4^ Institute of Biological Chemistry, Biophysics and Bioengineering, Heriot Watt University, Edinburgh EH14 4AS, UK; ^5^ Université Paris Cité, 75014 Paris, France; ^6^ Institut Cochin, Cancer Research Axis, INSERM U1016, 75014 Paris, France; ^7^ CNRS, UMR8104, 75014 Paris, France; ^8^ MRC Laboratory of Molecular Biology, Cambridge CB2 0QH, UK; ^9^ Research Department of Pathology, University College London, London WC1E 6DE, UK; ^10^ Cellular and Molecular Pathology, Royal National Orthopaedic Hospital, Stanmore HA7 4LP, UK; ^11^ Wolfson Diabetes and Endocrine Clinic, Addenbrooke's Hospital, Cambridge University Hospitals NHS Foundation Trust, Cambridge CB2 0QQ, UK; ^12^ Department of Genomic Medicine, National Institute for Health Research Cambridge Biomedical Research Centre, University of Cambridge CB2 0QQ, Cambridge, UK; ^13^ Wessex Clinical Genetics Service, University Hospital Southampton, Princess Anne Hospital, Southampton SO16 5YA, UK; ^14^ Human Development and Health, Faculty of Medicine, University of Southampton, Southampton SO16 5YA, UK; ^15^ Exepathology, Exmouth, UK

**Keywords:** PTEN, PI3K, PHTS, Cancer, Macrocephaly, ASD

## Abstract

PTEN hamartoma tumour syndrome (PHTS), a rare disease caused by germline heterozygous *PTEN* variants, is associated with multi-organ/tissue overgrowth, autism spectrum disorder and increased cancer risk. Phenotypic variability in PHTS is partly due to diverse *PTEN* variants and the protein's multifaceted functions. PTEN is primarily a phosphatidylinositol(3,4,5)trisphosphate (PIP_3_) phosphatase regulating PI3K/AKT signalling but also maintains chromosomal stability through nuclear functions such as double-stranded (ds)DNA damage repair. Here, we show that *PTEN-R173C*, a pathogenic variant frequently found in PHTS and somatic cancer, has elevated PIP_3_ phosphatase activity that effectively regulates canonical PI3K/AKT signalling. However, PTEN-R173C is unstable and excluded from the nucleus. We generated *Pten^+/R173C^* mice which developed few tumours during their lifetime, aligning with normal PI3K/AKT signalling. However, they exhibited lymphoid hyperplasia, macrocephaly and brain abnormalities, associated with impaired nuclear functions of PTEN-R173C, demonstrated by reduced dsDNA damage repair. We integrated PHTS patient data with our mouse model results, and propose that defective nuclear functions of *PTEN* variants can predict the onset of PHTS phenotypes and that late-onset cancer in these individuals may arise from secondary genetic alterations, facilitated by compromised dsDNA repair.

## Teaser

**Summary:** Germline heterozygous *PTEN* mutations are associated with tissue overgrowth, autism spectrum disorder and increased cancer risk; this study presents a mouse model representing recurrent human *PTEN* variants, to elucidate disease outcomes.

## INTRODUCTION

PTEN is a ubiquitously expressed tumour suppressor that shows complete loss of expression in some cancers (e.g. prostate cancer and glioblastoma) and over-representation of missense variants in others (e.g. endometrial cancer) (Alvarez-Garcia et al., 2019; Li et al., 1997; Steck et al., 1997; Yehia et al., 2020). Heterozygous germline variants of *PTEN* cause PTEN hamartoma tumour syndrome (PHTS), a rare autosomal dominant disorder. Recent estimates puts the prevalence of PHTS at 1:8000-13,000 (White et al., 2025), 10- to 20-fold higher than previous estimates (Nelen et al., 1996). Clinical features of PHTS include macrocephaly, developmental delay (DD), autism spectrum disorder (ASD), dermatological pathologies, gastrointestinal (GI) polyps, immune dysfunction and vascular anomalies. Individuals with PHTS also face a significantly increased risk of developing cancers, particularly of the breast, endometrium and thyroid (Ngeow and Eng, 2019; Yehia and Eng, 2018), with emerging evidence for non-PHTS cancers such as ovarian and prostate cancer (Schei-Andersen et al., 2025; Yehia et al., 2025).

PTEN predominantly acts as a lipid phosphatase metabolising phosphatidylinositol(3,4,5)trisphosphate (PIP_3_) and phosphatidylinositol(3,4)bisphosphate [PI(3,4)P_2_], thereby antagonising PI3K/AKT signalling (Myers et al., 1998). PTEN also has protein phosphatase activity, reducing phosphorylation of Ser/Thr/Tyr on heterologous protein substrates (Leslie et al., 2009), in addition to auto-dephosphorylating its T366 site (Tibarewal et al., 2012). Nuclear PTEN has been implicated in cell cycle control, double-stranded (ds)DNA repair, maintenance of chromatin structure, genome integrity and transcriptional control (Ho et al., 2019; Langdon, 2023).

The functional core of PTEN consists of a catalytic [amino acids (AA) 16-185] and a C2 (AA 186-350) domain (Lee et al., 1999), with mutations differentially impacting PTEN expression and function. Sixty-five percent of patients with PHTS express *PTEN* variants such as deletions, DNA insertions or non-sense variants leading to STOP codons (such as R130X and R233X), resulting in PTEN truncation, which are therefore predicted to cause heterozygous loss of expression. The remaining carry heterozygous missense variants in the phosphatase or C2 domain, leading to altered PTEN function or subcellular distribution (Hendricks et al., 2022; Spinelli et al., 2015). These include variants in the catalytic pocket, resulting in loss of all catalytic activity [e.g. C124 (C124S/R), R130 (R130G/Q/L) and G129 (G129R)] (Lee et al., 1999) or selective loss of PIP_3_ phosphatase activity, while retaining protein phosphatase activity (e.g. G129E) (Myers et al., 1998). Missense variants leading to selective loss of protein phosphatase activity (Y138L/C) have also been described, although these have not been reported in PHTS (Davidson et al., 2010; Tibarewal et al., 2012). Recent reports have shown that a subset of PTEN variants in PHTS (including K289E, D252G, F241S, I101T and others) are excluded from the nucleus and therefore lack PTEN nuclear functions (Langdon, 2023).

The PIP_3_ phosphatase activity of PTEN has long been regarded as critical in PHTS pathogenesis, yet other functions of PTEN are now also emerging as important. Various studies have attempted to establish genotype-phenotype associations in PHTS. Limited patient data make it challenging to ascertain the impact of individual variants in driving patient phenotypes. This challenge has been partially overcome by classifying the variants based on their position in the *PTEN* gene and their impact on PTEN expression and function (Lachlan et al., 2007; Leslie and Longy, 2016; Marsh et al., 1998, 1999; Mighell et al., 2018). Recently, a large paediatric and adult patient cohort study revealed that missense variants are associated with early disease onset, with DD and macrocephaly being the reason for early diagnosis, whereas *PTEN* truncation variants are associated with late onset disease, with cancer being the reason for PHTS diagnosis at later stages in life (Hendricks et al., 2022). However, patient cohort studies have been limited by the variability in age and sex of the individuals, one of the key challenges in rare disease research.

Genetically modified mice have helped overcome some of the challenges in PHTS research by allowing assessment of PTEN functions in an endogenous tissue context (Lee and Pandolfi, 2020). They also serve as a platform for testing potential treatments, such as PI3K pathway inhibitors, aimed at mitigating the effects of PTEN loss. An example are mice with heterozygous loss of PTEN expression (*Pten^+/−^*), representing *PTEN* variants that result in loss of expression and function. This mouse model recapitulates many aspects of human PHTS remarkably well in terms of cancer development and lymphoid and brain overgrowth (Podsypanina et al., 1999; Tibarewal et al., 2022).

Several mouse models have been developed to distinguish the relative importance of the cytosolic versus nuclear functions of PTEN (Langdon, 2023). For instance, *Pten^m3m4^* mice, feature non-naturally occurring *Pten* mutations that result in a predominantly cytosolic PTEN distribution without affecting its PIP_3_ phosphatase activity. These mice have been extensively studied for the impact of the nuclear-cytoplasmic partitioning of PTEN on neuronal and immune phenotypes, although their tumour spectrum has not been reported (Mester et al., 2011; Tilot et al., 2014; Jaini et al., 2020). Other models, expressing *PTEN* missense variants found in PHTS such as F341V (Caserta et al., 2015), and non-pathogenic mutations such as K13R, Y240F and D384V (Igarashi et al., 2018; Ma et al., 2019), that do not affect the catalytic functions of PTEN but impact its nuclear/sub-cellular localisation, have also been developed, but there are limited or no patient data for these individual variants (Table 1; Table S1).

**Table 1.** Clinical phenotypes of patients with PHTS with nuclear-excluded *PTEN* variants and *PTEN-R173* variants

		Patients with PHTS with nuclear-excluded *PTEN* variants	Patients with PHTS with *PTEN-R173* variants	Both groups combined
Patient demographics (*N*)	Number of patients	25	34	59
	Age range (median age)	1 year to 40 years (6 years)	7 months to 63 years (12.5 years)	7 months to 63 years (10 years)
	Sex distribution: *N* (%)	Males: 12 (48%) Females: 9 (36%) NR: 4 (16%)	Males: 19 (56%) Females: 14 (41%) NR: 1 (3%)	Males: 31(52%) Females: 23 (39%) NR: 5 (8%)
Phenotypes [*N* (%)]				
• Neuro-developmental	Macrocephaly	21 (84%)	32 (94%)	53 (90%)
	Developmental disorders	16 (64%)	18 (53%)	34 (58%)
• Cutaneous and vascular features	Cutaneous pathology	12 (48%)	16 (47%)	28 (47%)
	Vascular abnormalities	3 (12%)	5 (15%)	8 (14%)
	Lipomas	4 (16%)	−	4 (7%)
	Malignant cancer of skin	−	1 (3%) Age: 63 years	1 (1.7%)
• Thyroid	Benign lesions	4 (16%)	6 (18%)	10 (17%)
	Malignant cancer	1 (4%) Age: 29 years	1 (3%) Age: 59 years	2 (3%)
• Gastro-intestinal	Benign polyps	5 (20%)	4 (12%)	9 (15%)
• Breast	Benign lesions	1 (4%)	2 (6%)	3 (5%)
	Malignant cancer	−	2 (6%) Ages: 54 years and 59 years	2 (3%)
• Ovary	Benign lesions	−	3 (9%)	3 (5%)
	Malignant cancer	−	1 (3%) Age: 63 years	1 (1.7%)
• Uterus	Benign lesions	−	3 (9%)	3 (5%)
• Other cancers	Testicular cancer	1 (4%) Age: 29 years	−	1 (1.7%)
	Lung cancer	1 (4%) Age: NR	−	1 (1.7%)

NR, not recorded; PTHS, PTEN hamartoma tumour syndrome. Phenotypic categories include the following: developmental disorders (global developmental delay, delay in speech development, mental retardation, autism spectrum disorder, special educational needs), cutaneous pathology (hamartomas, tricholemmoma, mucocutaneous lesions, acral keratoses, palmoplantar keratoses, oral papillomas, penile freckling, facial papules, café-au-lait spots, skin tags), vascular abnormalities (haemangiomas, venous malformations, arterio-venous malformations), thyroid benign lesions (cyst, nodules, adenomas), thyroid malignant cancer (follicular cancer, papillary-follicular thyroid cancer), breast benign lesions (fibrocystic disease of the breast, fibroadenomas, benign breast disease), breast malignant cancer (ductal breast carcinoma, invasive ductal carcinoma and ductal carcinoma *in-situ* of the breast), ovary benign lesions (ovarian cysts, benign neoplasm), ovary malignant cancer (malignant neoplasm of the ovary) and uterus benign lesions (uterine fibroids, endometrial polyp). The phenotypes of individual patients are listed in Tables S1 and S2.

Here, we report the creation and characterisation of a novel mouse model for PHTS, carrying a *PTEN-R173C* [NM_000314.8(PTEN):c.517C>T (p.Arg173Cys)] missense variant in the germline. R173 is located in the Thr-Ile (TI) loop in the catalytic domain of PTEN important for catalytic activity. *PTEN-R173* variants, mainly *-R173C* and *-R173H* are commonly found in PHTS and in somatic cancers. We have identified 38 patients with PHTS with *PTEN-R173* variants (Table 1; Table S2). In somatic cancers, the PTEN-R173 site is also one of the three frequent mutational hot spots in *PTEN*, with 89 cases with R173C, 83 with R173H, two with R173P and R173L, and one case each with R173S and a silent mutation reported in the COSMIC database.

In this study, we first revisited the impact of this mutation on PTEN expression and function using biochemical and cell-based models and showed that PTEN-R173C has reduced protein stability but retains its ability to dephosphorylate PIP_3_ and therefore regulate the canonical PI3K/AKT pathway in the cytosol. However, we found that this PTEN mutant is largely excluded from the nucleus.

We created heterozygous germline *Pten^R173C^* knock-in mice (*Pten^+/R173C^*) as a unique model of a frequent pathogenic PHTS PTEN variant representing nuclear-excluded PTEN missense variants. We examined the consequences of this mutation on embryonic development, glucose metabolism and development of PHTS-relevant cancers, as well as immune, neurological and behavioural phenotypes. By integrating our findings with phenotypic data from patients for this variant, and by comparison with reported data on the *Pten^m3m4^* mice, we delineate the role of the nuclear functions of PTEN in the manifestation of PHTS phenotypes.

## RESULTS

### Increased PIP_3_ phosphatase activity but reduced protein stability of PTEN-R173 mutant proteins upon transient expression in mammalian cells

Conflicting data on the catalytic activity of *PTEN-R173* variants have been published (Han et al., 2000; Post et al., 2020; Smith and Briggs, 2016). PTEN-R173 mutant proteins, expressed in *Escherichia coli*, have been reported to be catalytically inactive (Han et al., 2000); however, mutant PTEN proteins expressed in non-mammalian systems often fail to fold properly, resulting in lack of activity (Spinelli et al., 2015). Consistent with this, in our hands, PTEN-R173C protein expressed in *E. coli* lacked *in vitro* PIP_3_ lipid phosphatase activity (Fig. S1A) and, when expressed in Sf9 insect cells (Masson et al., 2016a), precipitated upon purification, rendering it unsuitable for use in *in vitro* assays. Affinity purified GST-PTEN-R173C after transient expression in mammalian Lenti-X™ 293T cells possesses *in vitro* catalytic activity against soluble PIP_3_, albeit lower than PTEN-WT (Fig. S1B; WT, wild type). Although this assay validates PTEN-R173C as a bona fide PIP_3_ phosphatase, these *in vitro* conditions limit the ability to fully recapitulate the complexity of plasma membrane-associated PIP_3_ metabolism. Crucially, it does not account for potential modulatory effects arising from PTEN interaction with the plasma membrane. We therefore decided to use cell-based assays that represent the natural lipid substrates for PTEN.

**Fig. 1.**
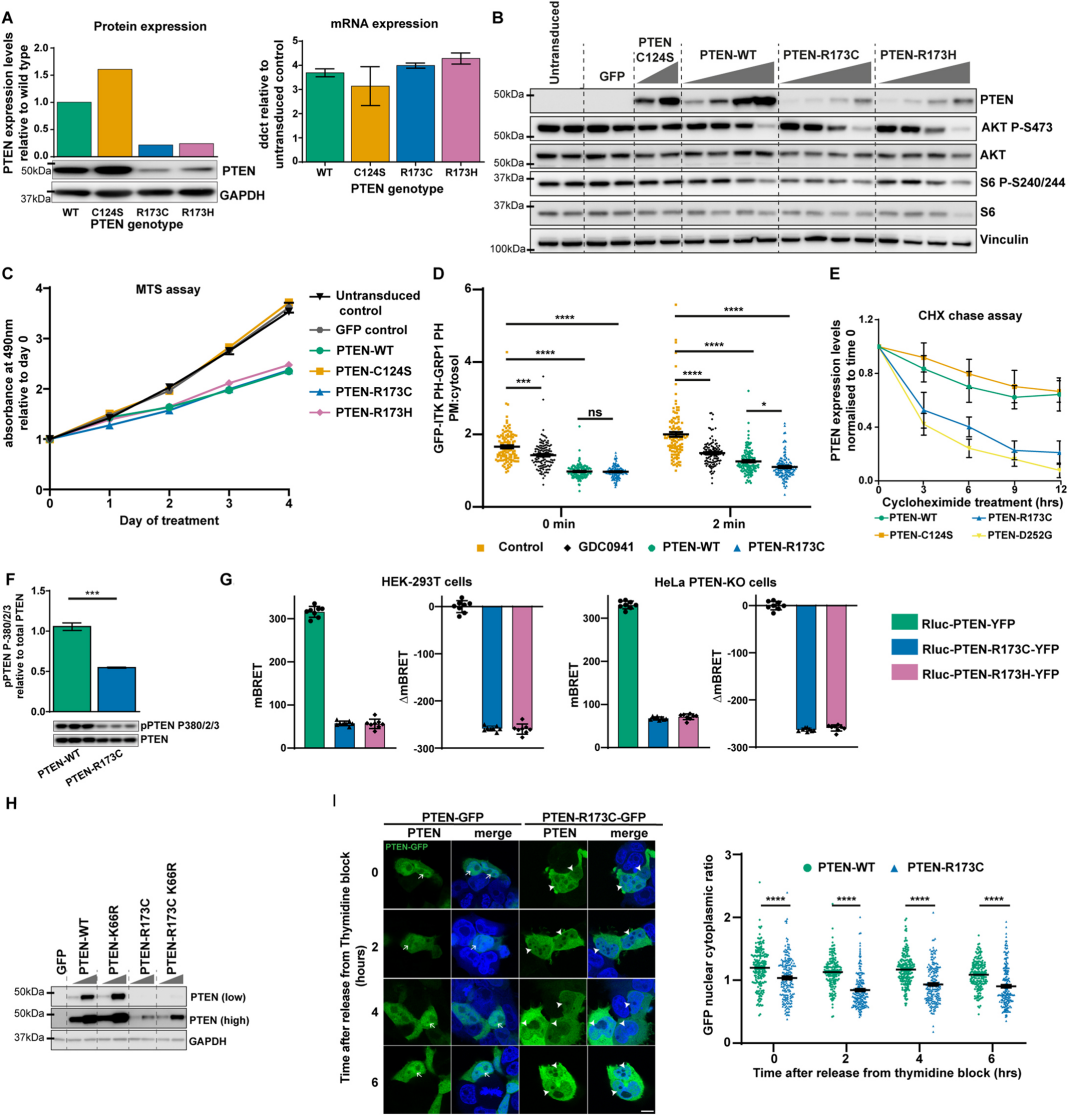
**Characterisation of PTEN-R173C/H.** (A-F,H) Transient lentiviral expression in U87 cells of PTEN-WT or mutants at fixed (A,C-F) or increasing concentrations of lentiviral particles (B,H). WT, wild type. (A, right) Quantitative PCR analysis was performed to determine *PTEN* mRNA levels (mean±s.e.m., *n*=3, right). (A, left; B) PTEN (A, left) and PTEN, AKT P-S473 and S6 P-S240/244 (B) expression assessed by immunoblotting. B shows a representative immunoblot from *n*=3. (C) Cell proliferation as determined by MTS assay over 4 days. Graph shows the mean±s.e.m. of absorbance at 490 nm relative to that on day 0 from ten replicate wells of a representative experiment from *n*=2. (D) U87 cells expressing a phosphatidylinositol(3,4,5)trisphosphate (PIP_3_) biosensor, with or without PTEN-WT or PTEN-R173C, were pre-treated with or without GDC-0941 (1 µM) for 1 h, with or without insulin treatment (100 nM) for 2 min. Mean fluorescence intensity (MFI) of the PIP_3_ biosensor at the plasma membrane normalised to the MFI of the PIP_3_ biosensor in the cytosol shown as mean±s.e.m. from three independent experiments, analysed using two-way ANOVA. (E) Cells were treated with cycloheximide (CHX), and PTEN protein expression levels were determined by immunoblotting at the indicated time points. Graph shows PTEN protein levels normalised to time 0, data shown as mean±s.e.m., *n*=6. (F) Phosphorylation of PTEN on S380/T382/T383 was determined by immunoblotting. Graph shows mean±s.e.m., *n*=3, analysed using two-tailed unpaired *t*-test. (G) Milli bioluminescent resonance energy transfer (mBRET) and change in mBRET (ΔmBRET) values, obtained in live HEK-293T and HeLa PTEN knockout (KO) cells, transfected with Rluc-PTEN-WT-YFP, Rluc-PTEN-R173C-YFP or Rluc-PTEN-R173H-YFP. Graphs represent mean±s.d. of *n*=8 points from a representative experiment from three independent experiments. (H) Protein extracts from U87 cells were used for immunoblotting to assess PTEN protein levels. (I) Lenti-X™ 293T cells expressing GFP-tagged PTEN-WT or PTEN-R173C were arrested in the G1/S phase of the cell cycle by double-thymidine block and imaged by confocal microscopy at the indicated time points after release from thymidine block, for expression of PTEN-GFP. Left panel shows representative images. Arrows indicate nuclear PTEN-WT; arrowheads indicate reduced nuclear PTEN-R173C. Scale bar: 10 µm. Graph shows the nuclear:cytoplasmic ratio of the MFI of the PTEN-GFP signal (mean±s.e.m.; *n*=3, analysed using two-way ANOVA followed by Tukey's multiple comparison test). *P<0.05; ***P<0.001; ****P<0.0001; ns, not-significant (*P*>0.05).

A commonly used assay to determine the ability of PTEN and its mutants to regulate PI3K/AKT signalling involves expression in the PTEN-null U-87 MG glioblastoma cell line (further referred to as U87) and assessment of AKT phosphorylation and cell proliferation (Davidson et al., 2010). Transient lentiviral expression of untagged PTEN-R173C in U87 revealed consistently lower protein expression levels compared to those for PTEN-WT (Fig. 1A, left), despite similar mRNA expression levels (Fig. 1A, right). Similar results were seen with PTEN-R173H.

Expression of a dose range of PTEN proteins in U87 revealed that, compared to PTEN-WT, lower levels of PTEN-R173C or PTEN-R173H effectively downregulated AKT P-S473 phosphorylation (Fig. 1B; Fig. S1C) and suppressed U87 proliferation (Fig. 1C; Fig. S2A). We also assessed the impact of PTEN-R173C on recruitment of a PIP_3_-biosensor protein to the plasma membrane in U87 cells. In insulin-stimulated cells, the biosensor plasma membrane to cytosol ratio (plasma membrane:cytosol) was significantly reduced upon treatment with the pan-class I PI3K inhibitor GDC-0941 or upon expression of PTEN-WT or PTEN-R173C, with the strongest decrease upon PTEN-R173C expression (Fig. 1D; Fig. S2B), despite its lower expression levels compared to those for PTEN-WT (Fig. S2C).

Taken together, these data suggest that, in mammalian cells, PTEN-R173C is less stable but catalytically more active than PTEN-WT.

### Evidence for a more open, proteolytically sensitive protein conformation of PTEN-R173 mutants

We next investigated possible biochemical mechanisms underlying the reduced expression of PTEN-R173C. Cycloheximide chase experiments of U87 cells transiently transduced with PTEN expression lentiviruses indicated a reduced protein half-life of PTEN-R173C compared to that of PTEN-WT (Fig. 1E; Fig. S3A). A recent study has shown similar findings with PTEN-R173H (Jun et al., 2024).

PTEN protein stability and activity is regulated, in part, by phosphorylation on multiple C-terminal sites (S380/T382/T383/S385) by CK2, resulting in a more closed and stable but less active protein conformation. Dephosphorylation releases the inhibitory effect of these phosphorylated residues on the C2 domain of PTEN, leading to a more open conformation with increased membrane binding and therefore enhanced activity (Torres and Pulido, 2001; Vazquez et al., 2001; Birle et al., 2002; Odriozola et al., 2007; Dempsey et al., 2021). Once at the membrane, the active form of PTEN becomes degraded (Maccario et al., 2010). Upon transient expression of similar levels of untagged proteins in U87 cells, PTEN-R173C showed a ∼50% lower level of S380/T382/T383 phosphorylation compared to PTEN-WT (Fig. 1F). Similar data were observed using YFP-tagged Rluc-PTEN-R173C and PTEN-R173H in HEK-293T cells (Fig. S3B), suggesting that reduced phosphorylation contributes to the observed instability of PTEN-R173 mutants.

A study using computational modelling predicted increased protein flexibility of PTEN-R173C/H mutants, leading to impaired protein stability (Smith and Briggs, 2016). In order to gain insight into these phenomena, we used an intramolecular bioluminescent resonance energy transfer (BRET)-based biosensor Rluc-PTEN-YFP in live HEK-293T and PTEN knockout HeLa cells. This biosensor reveals dynamic changes in PTEN conformational rearrangement and function due to shifts in energy transfer between the donor/acceptor couple (Lima-Fernandes et al., 2014) (Fig. S4A,B). Using this approach, PTEN-R173C and PTEN-R173H were found to be in a more open/flexible conformation compared to PTEN-WT (Fig. 1G).

We previously reported that PTEN protein dephosphorylated on S380/T382/T383/S385 is more prone to ubiquitination at Lys66 and degradation (Maccario et al., 2010; Gupta and Leslie, 2016). In line with this, when transiently expressed in U87 cells, the PTEN-R173C/K66R double mutant, which cannot be ubiquitinylated, showed increased protein expression levels compared to PTEN-R173C (Fig. 1H).

Altogether, these data indicate that the PTEN-R173C protein has a more open conformation that allows ubiquitination and degradation, leading to reduced protein levels.

### PTEN-R173 mutants are predominantly cytoplasmic and largely excluded from the nucleus

The tumour suppressor functions of PTEN have been mostly attributed to its ability to regulate the PI3K/AKT pathway, with the role of other PTEN functions such as protein phosphatase activity and nuclear functions less well studied in this context (Ho et al., 2019). Given that our results so far showed that PTEN-R173C/H mutants are effective at regulating the PI3K/AKT pathway, we investigated other PTEN functions to explain the high mutation frequency of this site in somatic cancers and PHTS.

To assess potential alterations in the intrinsic protein phosphatase activity of the PTEN-R173C variant, we quantified phosphorylation levels at threonine 366 (Thr366), a known auto-dephosphorylation site (Tibarewal et al., 2012). Comparative analysis revealed no significant difference in Thr366 phosphorylation between GFP-tagged PTEN-R173C and PTEN-WT, indicating normal protein phosphatase activity (Fig. S5A).

We next analysed subcellular distribution of PTEN-R173C by transiently expressing C-terminally GFP-tagged PTEN constructs in Lenti-X™ 293T cells. We observed a significantly lower nuclear:cytoplasmic ratio of the GFP signal in cells expressing PTEN-R173C-GFP than in cells with PTEN-WT-GFP, suggesting that PTEN-R173C was largely excluded from the nucleus (Fig. 1I). Nuclear exclusion of the R173 mutants was also revealed upon expression of YFP-tagged Rluc-PTEN-WT, -R173C or -R173H in HEK-293T or PTEN knockout HeLa cells (Fig. S5B).

### Homozygous germline PTEN-R173C expression is embryonically lethal in mice

**Fig. 2.**
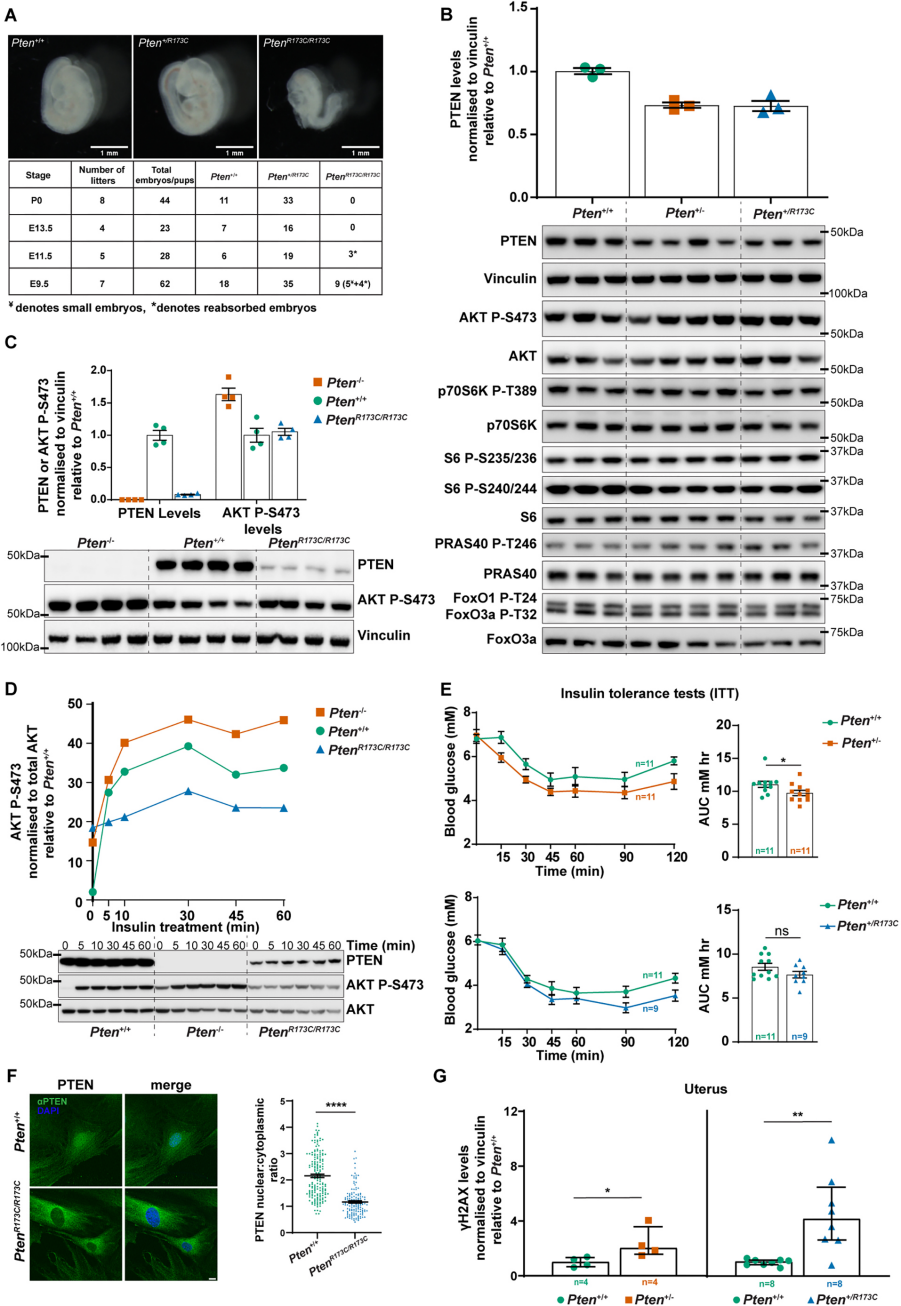
**Characterisation of *Pten^+/R173C^* mice.** (A) Images show morphology of embryonic day (E)9.5 *Pten^+/+^*, *Pten^+/R173C^* and *Pten^R173C/R173C^* embryos. Scale bars: 1 mm. Table shows the number of embryos/pups of each genotype obtained at the indicated time points. ‘¥’ indicates embryos smaller in size and ‘*’ indicates embryos that had been reabsorbed. (B,C) Protein extracts from mouse embryonic fibroblasts (MEFs) of the indicated genotype were used for immunoblotting with the antibodies shown. Data shown as mean±s.e.m. At least three independent MEF lines of each were used for B and two independent MEF lines were used for C. (D) MEFs of the indicated genotype were treated with insulin (100 nM) for the indicated times, and protein extracts were immunoblotted with the antibodies shown. Data shown as mean from two independent MEF lines for each genotype. (E) Insulin tolerance test assays on 3-month-old male mice of the indicated genotypes. *xy* curves show blood glucose levels post insulin injection over time. Bar graphs show mean±s.e.m. of measurements of area under the curve (AUC), analysed using two-tailed unpaired *t*-test. (F) MEFs of the indicated genotype were arrested in the G1/S phase of the cell cycle by double-thymidine block, immunostained with antibodies against PTEN (green) and DAPI (blue), and imaged by confocal microscopy 6 h after release from thymidine block. Scale bar: 10 µm. Graphs show nuclear:cytoplasmic ratio of the GFP MFI (mean±s.e.m. from three independent experiments) analysed using two-tailed unpaired *t*-test. (G) 6-week-old mice of the indicated genotypes were treated with 7 Gy γ-radiation, and protein extracts from the uterus were used for immunoblotting. Graph shows levels of γH2AX relative to those of littermate *Pten*^+/+^ controls as median with interquartile range analysed using Mann–Whitney *U*-tests. *P<0.05; **P<0.01; ****P<0.0001; ns, not-significant (*P*>0.05). Mice used for generating MEF lines in B-D and F, and in E and G, were on a C57BL/6J background.

To study the role of endogenously expressed PTEN-R173C in PHTS, we created heterozygous germline *Pten^R173C^* knock-in mice (*Pten^+/R173C^*) by CRISPR-Cas9 technology (Fig. S6). Intercrosses of *Pten^+/R173C^* mice did not yield live-born homozygous *Pten^R173C/R173C^* mice (Fig. 2A). *Pten^R173C/R173C^* embryos were reabsorbed at embryonic day (E)11.5, with under-representation compared to Mendelian ratios at earlier time points (Fig. 2A). Intact *Pten^R173C/R173C^* embryos were found at E9.5, with 5/9 embryos smaller and that had not undergone turning, with the remaining 4/9 reabsorbed, suggesting that *Pten^R173C/R173C^* embryos die around E9.5 (Fig. 2A).

Given the non-viability of homozygous *Pten^R173C/R173C^* mice, and the fact that *PTEN* variants in PHTS individuals are heterozygous, we used heterozygous *Pten^+/R173C^* mice for further investigation. For these studies, we used *Pten^+/−^* mice, a well-characterised model of PHTS (Knobbe et al., 2008; Di Cristofano et al., 1998, 2001; Suzuki et al., 1998; Podsypanina et al., 1999; Freeman et al., 2006), alongside littermate WT (*Pten^+/+^*) mice as controls.

### PTEN-R173C expressed from its endogenous promoter is unstable but retains PIP_3_ phosphatase activity

To study endogenous PTEN-R173C protein expression and function, we derived mouse embryonic fibroblasts (MEFs) of the genotypes *Pten^+/R173C^*, *Pten^R173C/R173C^* and *Pten^−/R173C^*, and compared these to *Pten^+/−^*, *Pten^−/−^* and *Pten^+/+^* MEFs.

PTEN protein expression levels in *Pten^+/R173C^* and *Pten^+/−^* MEFs were 60-70% of those in *Pten*^+/+^ MEFs (Fig. 2B). The PTEN levels observed in *Pten*^+/−^ MEFs agree with previous studies in which the *Pten^WT^* allele was shown to express higher than 50% level of PTEN protein (Alimonti et al., 2010). In *Pten^R173C/R173C^* and *Pten^−/R173C^* MEFs, PTEN protein levels were ∼13% and ∼2%, respectively, of those in *Pten*^+/+^ MEFs (Fig. 2C; Fig. S7), indicative of low protein stability of the PTEN-R173C protein, in line with our exogenous expression studies above.

We next assessed PI3K pathway activation, under standard cell culture conditions and upon insulin stimulation. *Pten^+/R173C^*, *Pten*^+/−^ and *Pten*^+/+^ MEFs growing in complete media showed similar basal levels of phosphorylation of AKT, S6 and other downstream effectors (Fig. 2B). Under these conditions, *Pten*^−/−^ MEFs had increased levels of AKT P-S473, which was not observed in *Pten^R173C/R173C^* MEFs (Fig. 2C). In *Pten^−/R173C^* MEFs, the levels of AKT P-S473 were elevated compared to those in *Pten^+/+^* and *Pten*^+/−^ MEFs, but still substantially lower than those in *Pten^−/−^* MEFs (Fig. S7).

Despite the very low levels of PTEN-R173C expression, upon insulin stimulation, there was no increase in AKT P-S473 above basal levels in *Pten^R173C/R173C^* MEFs, in contrast to increased AKT P-S473 observed in *Pten*^+/+^ and *Pten*^−/−^ MEFs (Fig. 2D).

Taken together, these data indicate that, even at low levels of expression, PTEN-R173C is still capable of effectively downregulating unstimulated and insulin-stimulated AKT P-S473 phosphorylation and thus retains overall PIP_3_ phosphatase activity.

### PTEN-R173C regulates glucose metabolism *in vivo*

PTEN plays an important role in glucose metabolism by dampening insulin signalling, with *Pten*^+/−^ mice showing enhanced acute insulin signalling and increased glucose metabolism (Chen et al., 2018; Garcia-Cao et al., 2012; Pal et al., 2012). Patients with PHTS also often show constitutive insulin sensitisation (Pal et al., 2012). In line with these reports, insulin tolerance tests on 3-month-old mice revealed enhanced insulin sensitivity in *Pten*^+/−^ mice, evidenced by lower levels of blood glucose post insulin injection compared to those in *Pten^+/+^* littermates (Fig. 2E). In contrast, *Pten^+/R173C^* mice showed a similar insulin response up to 60 min after insulin injection (Fig. 2E). This indicates that, at the organismal level, PTEN-R173C maintains the ability to downregulate acute insulin/PI3K/AKT signalling and therefore glucose homeostasis (Fig. 2E). However, from the 90 min time point onwards, *Pten^+/R173C^* mice showed slightly increased clearance of blood glucose compared to WT littermates (Fig. 2E). It is tempting to speculate that this might be due to recruitment of PTEN-R173C to the plasma membrane upon insulin stimulation and associated rapid degradation, with total PTEN protein levels becoming similar to those in *Pten^+/−^* tissues, resulting in an increased insulin sensitivity at these later time points.

### Endogenously expressed PTEN-R173C is predominantly nuclear excluded with reduced ability to regulate dsDNA damage repair

To determine the subcellular distribution of PTEN-R173C upon expression from its endogenous locus, we used immunocytochemistry and quantification of the PTEN nuclear/cytoplasmic ratio in *Pten^R173C/R173C^* and *Pten^−/R173C^* MEFs. These data showed that PTEN-R173C expressed from its endogenous locus was predominantly nuclear excluded, in contrast to PTEN-WT protein, which was present in both the nucleus and cytoplasm (Fig. 2F; Fig. S8A). Given that R173 is not located in a known PTEN nuclear localisation signal sequence, one reason for the apparent nuclear exclusion of PTEN-R173C could be sequestration at the plasma membrane, with little protein available for nuclear import. To investigate this, we treated *Pten^R173C/R173C^* MEFs with the pan-class I PI3K inhibitor GDC-0941, with the aim to reduce the PIP_3_ substrate levels for PTEN at the plasma membrane and to potentially restore the presence of PTEN-R173C in the nucleus. However, GDC-0941 treatment did not affect the subcellular distribution of PTEN-R173C (Fig. S8B), suggesting that plasma membrane PIP_3_ levels do not significantly contribute to the nuclear exclusion of PTEN-R173C.

Nuclear PTEN has numerous functions including regulation of DNA repair, transcription, chromatin structure, chromosomal stability and genome integrity by various mechanisms (Ho et al., 2019; Langdon, 2023). We investigated one of these nuclear functions in *Pten^+/R173C^* mice, namely dsDNA damage repair. Nuclear PTEN deficiency has been shown to cause a homologous recombination (HR) defect in cells, leading to increased γH2AX expression and decreased Rad51 foci formation upon genotoxic stress such as ionising radiation (IR) (Bassi et al., 2013; Mendes-Pereira et al., 2009; Shen et al., 2007). Analysis of *Pten^+/R173C^* and *Pten*^+/−^ mice, 5 h post-IR treatment, showed a significant increase in γH2AX levels in protein extracts from uteri, kidney and liver, compared to those in the same extracts from littermate WT mice (Fig. 2G; Fig. S9), indicative of a similarly reduced dsDNA repair capacity upon heterozygous PTEN loss or heterozygous PTEN-R173C expression. This suggests that PTEN-R173C has reduced ability to regulate DNA damage repair in these tissues compared to PTEN-WT.

### Reduced cancer predisposition of *Pten^+/R173C^* mice compared to *Pten^+/−^* mice

Individuals with PHTS have increased risk of cancers of multiple tissues including breast, endometrium and thyroid (Yehia and Eng, 2018; Yehia et al., 2020, 2019). To gain insight into the cancer predisposition of germline *Pten^R173C^* expression, we aged heterozygous *Pten^+/R173C^* mice alongside *Pten*^+/−^ and *Pten*^+/+^ littermate controls. We used mice on a mixed C57Bl/6JxSv129 background, as on this background *Pten^+/−^* mice develop PHTS-relevant phenotypes, with an overlapping tumour spectrum including hyperplasia/tumours of the thyroid, endometrium, lymphoid tissue, small intestine and adenomyoepithelioma, and malignant mammary tumours in the females (Tibarewal et al., 2022). *Pten*^+/−^ mice also develop lesions not seen in patients with PHTS including, pheochromocytoma and intraepithelial neoplasia (PIN) and adenocarcinoma of the prostate (Knobbe et al., 2008; Di Cristofano et al., 1998, 2001; Suzuki et al., 1998; Podsypanina et al., 1999; Freeman et al., 2006).

Gross morphology and development of *Pten^+/R173C^* mice was found to be similar to that of *Pten*^+/+^ littermates, with a tendency for a slight increase in body weight at a young age in both *Pten^+/R173C^* and *Pten*^+/−^ mice (Fig. S10).

**Fig. 3.**
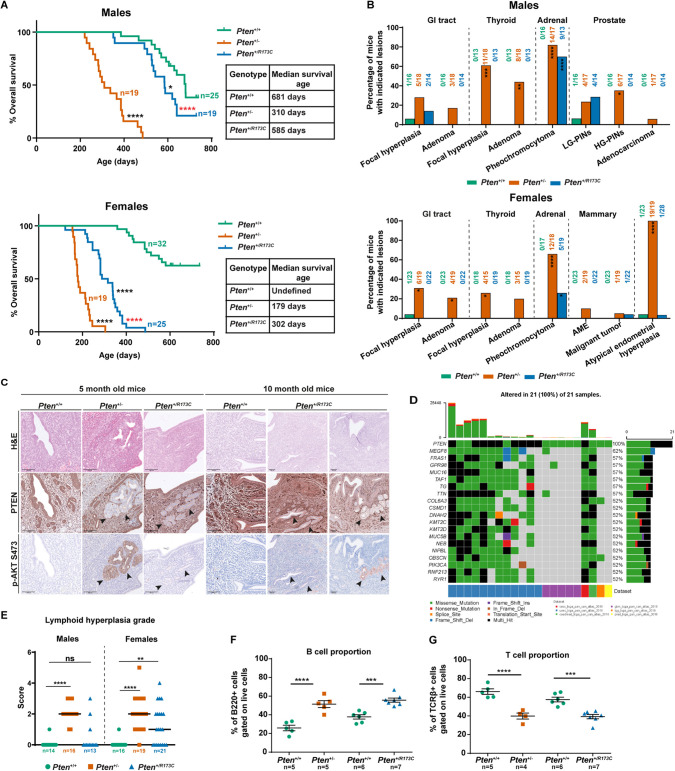
**Survival and histopathological analysis of *Pten^+/R173C^* mice.** Mice on a mixed C57BL/6J×Sv129 background were allowed to age and euthanised for welfare reasons or at a specified age. (A) Kaplan–Meier survival curves for male and female mice analysed using Log-rank (Mantel–Cox) test and Gehan–Breslow–Wilcoxon test. Comparisons were made to *Pten*^+/+^ mice (*P*-values shown as black asterisks) or *Pten*^+/−^ mice (*P*-values shown as red asterisks). Table shows median survival age of the mice. (B) Incidence of specific tumour types in male and female mice, as assessed by histopathological analysis of all mice, analysed using Fisher's exact test. AME, adenomyoepithelioma; GI, gastrointestinal; HG-PIN, high-grade pheochromocytoma and intraepithelial neoplasia; LG-PIN, low-grade pheochromocytoma and intraepithelial neoplasia. (C) Representative immunohistochemical images of uteri from female mice at the indicated timepoints; black arrowheads indicate loss of PTEN immunoreactivity and the corresponding AKT P-S473 immunoreactivity. H&E, Haematoxylin and Eosin. Scale bars: 100 µm. (D) Oncoplot showing co-occurrence of mutations in the indicated genes in tumour samples harbouring PTEN-R173C variant (cBioportal). (E) Scatter plot of lymphoid hyperplasia scores from individual mice at the end of study with line at median, analysed using Fisher's exact test. (F,G) Flow cytometry analysis was performed on the lymph nodes of female *Pten^+/R173C^*, *Pten*^+/−^ and littermate *Pten*^+/+^ controls at 5 and 10 months of age. Graphs show the proportion of B220^+^ B-cells (F) and TCRβ^+^ T-cells (G), analysed using one-way ANOVA . *P<0.05; **P<0.01; ***P<0.001; ****P<0.0001; ns, not-significant (*P*>0.05).

Mice were aged and sacrificed at first appearance of signs of ill health or palpable masses. The reasons for humane culling of mice are listed in Table S3. Under these experimental conditions, the median survival age of *Pten^+/R173C^* mice was 585 and 302 days for male and female mice, respectively. This is significantly longer than the median survival age of 310 and 179 days for male and female *Pten*^+/−^ mice, respectively (Fig. 3A). However, *Pten^+/R173C^* mice had a shorter lifespan than that of WT mice (Fig. 3A), especially female *Pten^+/R173C^* mice, who were mainly euthanised owing to palpable masses or ill health.

Histopathological analysis of selected tissues from *Pten*^+/−^ and *Pten^+/R173C^* mice revealed very similar gross morphologies of all tissues analysed, with overall histopathology data summarised in Fig. 3B and Tables S4-S7.

*Pten^+/R173C^* mice developed far fewer tumours than *Pten*^+/−^ mice, with occasional hyperplasia of the small intestine (2/14 mice) and low-grade PINs of prostate (4/14 mice), one case of mammary adenocarcinoma (1/22 mice) and one case of endometrial hyperplasia (1/28) (Fig. 3B; Figs S11 and S12, Tables S4 and S5). However, similar to *Pten*^+/−^ mice, *Pten^+/R173C^* mice had a high incidence of pheochromocytoma (Fig. 3B; Figs S11 and S12, Tables S4 and S5).

One out of 28 *Pten^+/R173C^* mice displayed a single focus of endometrial hyperplasia, with loss of immunostaining for PTEN and increased AKT P-S473 (Fig. 3C). Interestingly, loss of PTEN immunoreactivity was also observed in microscopically normal endometrial glands from 5-month-old and 10-month-old *Pten^+/R173C^* mice, but this was not accompanied by a concomitant increase in AKT P-S473 immunoreactivity in these regions (Fig. 3C). In the absence of reagents that allow us to specifically stain for WT or PTEN-R173C protein and, based on the low expression level of endogenous PTEN-R173C protein when expressed alone (Fig. S7), it is most likely that expression of the WT *Pten* allele is lost in these morphologically normal glands. We speculate that, although the very low expression level of PTEN-R173C from the endogenous allele may not be detectable by immunohistochemistry, it is still sufficient to partially downregulate PI3K signalling and AKT P-S473 and thereby prevent the development of endometrial tumours. Although this observation is based on a single mouse, we speculate that the hyperplastic gland found in the endometrium of this *Pten^+/R173C^* mouse may have resulted either from loss of expression of the remaining *Pten^R173C^* allele or from acquisition of PI3K pathway-activating mutations. These events likely led to PI3K pathway hyperactivation, ultimately contributing to the development of endometrial hyperplasia. This hypothesis is supported by the fact that somatic cancers with PTEN-R173 variants, along with mutation of the WT allele of *PTEN*, also contain mutations in other cancer drivers such as *PIK3CA* (Fig. 3D).

### Prominent but delayed lymphoid hyperplasia in *Pten^+/R173C^* mice compared to *Pten*^+/−^ mice

Previous studies have reported a range of lymphoid and immune phenotypes in patients with PHTS including peripheral lymphoid hyperplasia, enlargement of tonsils and adenoids, hypogammaglobulinemia, recurrent respiratory tract infection and autoimmune disorders (Chen et al., 2017; Drissen et al., 2024). Some of these phenotypes are found in patients with PHTS with *PTEN-*R173 variants and other nuclear-excluded variants (Tables S1 and S2).

In *Pten* mutant mice, including *Pten^+/m3m4^* mice, lymphoid hyperplasia leads to enlarged cervical, brachial, inguinal and axillary lymph nodes (Podsypanina et al., 1999; Alimonti et al., 2010; Jaini et al., 2020). Although this does not cause discomfort to the mice, in our study it was a main reason for sacrificing *Pten*^+/−^ mice (75% males and 80% females) and many *Pten*^+/R173C^ mice (21% males and 50% females), owing to animal welfare regulations on the allowed tissue overgrowth size (Table S3).

Histological analysis revealed a similar severity grade of hyperplasia (assessed by semi-quantitative histopathological scoring) in female *Pten^+/R173C^* and *Pten*^+/−^ mice but of significantly lower grade in *Pten^+/R173C^* males than in *Pten*^+/−^ males (Fig. 3E).

Flow cytometry analysis of lymph nodes of 5-month-old female *Pten*^+/−^ and 10-month-old female *Pten^+/R173C^* mice, the age at which they have visibly enlarged lymph nodes, compared to those of littermate *Pten*^+/+^ mice, revealed a significant increase in the proportion of B-cells and a corresponding decrease in T-cell proportions (Fig. 3F,G), a phenotype also reported in *Pten^+/m3m4^* mice (Jaini et al., 2020).

### Macrocephaly, increased neuronal size and white matter disturbances in *Pten^+/R173C^* mice

Patients with PHTS, including the majority of patients with PTEN-R173 variants (Table S2), commonly present with macrocephaly, DD and ASD. We therefore explored neurological and behavioural phenotypes in *Pten^+/R173C^* mice.

We examined the brains and neurons from *Pten*^+/+^, *Pten^+/R173C^* and *Pten*^+/−^ mice and, where possible, conditional knockout mice that selectively lack PTEN expression in cortical pyramidal neurons (referred to here as cKO) (Kessaris et al., 2006; Lesche et al., 2002). These cKO mice were further crossed to *Pten^+/R173C^* mice to obtain conditional knock-in mice (referred to here as cKI), which express PTEN-R173C in cortical pyramidal neurons without a copy of the PTEN-WT allele and hence are overall hemizygous for the *Pten^R173C^* allele (*Pten^−/R173C^*). The observed phenotypes are summarised in Table 2, which also shows a comparison to phenotypes published for *Pten^m3m4^* mice. The latter models germline expression of non-naturally occurring PTEN mutations that leave PTEN PIP_3_ phosphatase activity intact, but leads to its nuclear exclusion, and the neuronal phenotypes of which have been extensively characterised (Mester et al., 2011; Tilot et al., 2014; Jaini et al., 2020).

**Table 2.** Neuronal and behavioural phenotypes in mice with *Pten* mutations

Phenotypes	Mouse genotype				
	*Pten^+/R173C^*	*Pten^+/−^*	*Pten^−/−^*	*Pten^+/m3m4^*	*Pten^m3m4/m3m4^*
Increased brain size	Yes	Yes	NT	Yes	Yes
Bigger corpus callosum	Yes	Yes	NT	NT	NT
Increased numbers of oligodendroglia cells	Yes	No	NT	No	Yes
Astrogliosis	No	No	NT	No	Yes
Increased cell body size	Yes	No	Yes	No	Yes
Increased neuronal complexity	No	No	Yes	No	No
Enlarged axons	Yes	Yes	NT	NT	NT
Thicker myelin	Yes	Yes	NT	NT	NT
Sociability	No significant difference	NT	NT	No significant difference	Too social according to revised criteria
Motor coordination	NT	NT	NT	NT	Reduced
Locomotion	Reduced	NT	NT	NT	NT

Table shows data from *Pten^+/R173C^*, *Pten^+/−^* and conditional *Pten^−/−^* mice from this study, and *Pten^+/m3m4^* and *Pten^m3m4/m3m4^* mice reported previously (Mester et al., 2011; Tilot et al., 2014). NT, not tested. The behavioural assay (sociability, motor coordination and locomotion) results are relative to those from wild-type littermate mice.

We first assessed the levels of PTEN protein and downstream pathways in protein lysates from cortical neurons. In the absence of a PTEN-WT allele, the level of PTEN protein in cKI was minimal and similar to that seen in protein extracts of cKO mice (Fig. S13A,B). However, the level of AKT P-S473 in cKI brains was significantly lower than that in cKO brains (Fig. S13A,C). These observations mirror our data in MEFs (Fig. S7), indicating that PTEN-R173C is also highly active in the brain despite its low level of expression.

**Fig. 4.**
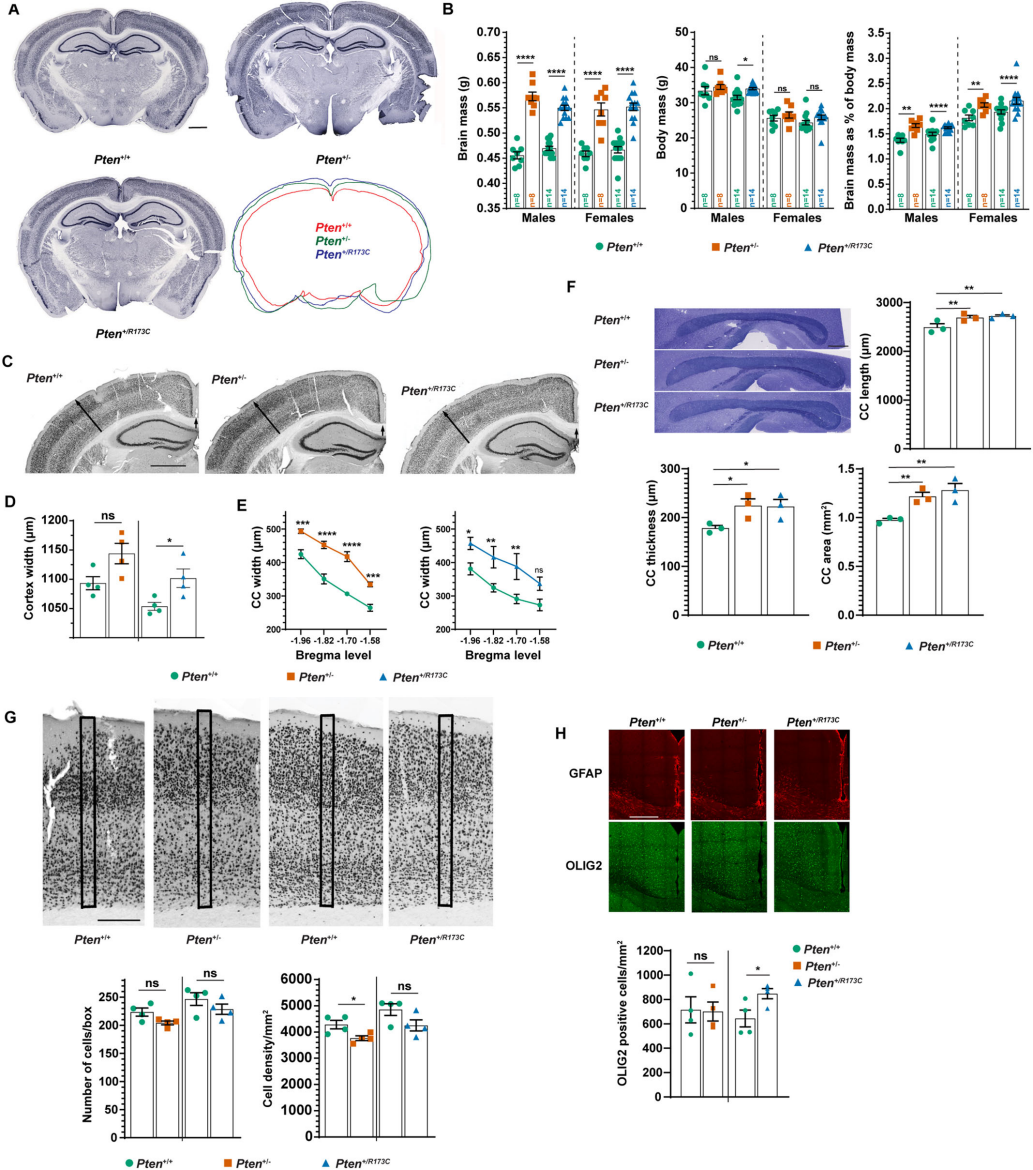
**Enlarged brain phenotype with associated changes in grey and white matter areas in *Pten* mutant mice.** (A) Representative NeuN immunostaining of coronal sections. The overlay of the contours of the depicted slices is also shown. Scale bar: 1000 µm. (B) Brain mass, body mass and brain mass as a percentage of body mass shown as mean±s.e.m., analysed using two-way ANOVA followed by Tukey's multiple comparison test. (C) Representative NeuN immunostaining from coronal sections. Scale bar: 1000 µm. (D) Quantification of cortical thickness in the primary somatosensory cortex barrel field (area indicated with long arrows in C) from *n*=4 mice of each sex and genotype shown as mean±s.e.m., analysed using two-tailed unpaired *t*-test. (E) Quantification of the thickness of the corpus callosum (CC) at the indicated Bregma levels (area indicated with short arrows in C) from *n*=4 mice of each sex and genotype, shown as mean±s.e.m., analysed using two-way repeated measures (RM) ANOVA with uncorrected Fisher's LSD test. (F) Top left: representative sagittal sections from mouse brains at Bregma level stained with Toluidine Blue. Scale bar: 500 µm. Quantification of the corpus callosum length (top right), thickness (bottom left) and area (bottom right) from *n*=3 mice per genotype shown as mean±s.e.m., analysed using one-way RM ANOVA followed by uncorrected Fisher's LSD test. (G) Top: representative images of coronal sections from the somatosensory cortex stained for NeuN. Scale bar: 250 µm. Bottom: quantification of the number and density of neurons in the indicated boxed areas in the top panel, from *n*=4 mice of each sex of each genotype shown as mean±s.e.m., analysed using two-tailed unpaired *t*-test. (H) Top: representative images of sections immunostained with OLIG2 and GFAP. Scale bar: 100 µm. Bottom: quantification of OLIG2-positive cells in the motor cortex from *n*=4 mice per genotype shown as mean±s.e.m., analysed using two-tailed unpaired *t*-test. *P<0.05; **P<0.01; ***P<0.001; ****P<0.0001; ns, not-significant (*P*>0.05). Mice used were 3-month-old on a mixed C57BL/6J×Sv129 background.

At 3 months of age, *Pten^+/R173C^* and *Pten*^+/−^ mice of both sexes had enlarged brains with increased mass compared to those of *Pten*^+/+^ littermates (Fig. 4A,B), indicating a macrocephalic phenotype. Also, at 3 and 6 months of age, and independent of the genetic background, *Pten^+/R173C^* and *Pten*^+/−^ mice showed a similar increase in brain mass (Fig. S14). Given the apparently normal PI3K/AKT regulatory capacity of PTEN-R173C, and the neurological phenotypes and macrocephaly previously observed in nuclear-excluded PTEN in heterozygous *Pten^+/m3m4^* and homozygous *Pten^m3m4/m3m4^* mice (Mester et al., 2011; Tilot et al., 2014), these observations allow us to postulate that the nuclear function of PTEN is key to the development of macrocephaly.

We next quantified several brain parameters to characterise the macrocephalic phenotype. *Pten^+/R173C^* mice had increased cortical thickness as measured at the level of the somatosensory barrel field (Fig. 4C,D). A significant increase was also observed in the thickness of the corpus callosum at four different Bregma levels (Fig. 4E) in both *Pten^+/R173C^* and *Pten*^+/−^ mice. Consistent with macrocephaly, lengthwise enlargement of the corpus callosum at Bregma levels together with an increase in the thickness and total area were also observed (Fig. 4F). The increased thickness of the cortex was not caused by increased neuronal cell numbers or neuronal densities in *Pten^+/R173C^* mice (Fig. 4G). In contrast, immunohistochemistry for OLIG2 revealed a small, but significant, increase in oligodendrocyte lineage cell numbers (Fig. 4H). Interestingly, this increase was not seen in our *Pten^+/−^* mice, and has not been reported in *Pten^+/m3m4^* mice, but was observed in *Pten^m3m4/m3m4^* mice (Table 2). Astrocyte overabundance and astrogliosis, as detected by GFAP immunostaining, were not observed (Fig. 4H). However, astrogliosis has been reported in *Pten^m3m4/m3m4^* mice (Table 2), suggesting that the remaining WT *Pten* allele protects the brain from this immunoinflammatory phenotype.

**Fig. 5.**
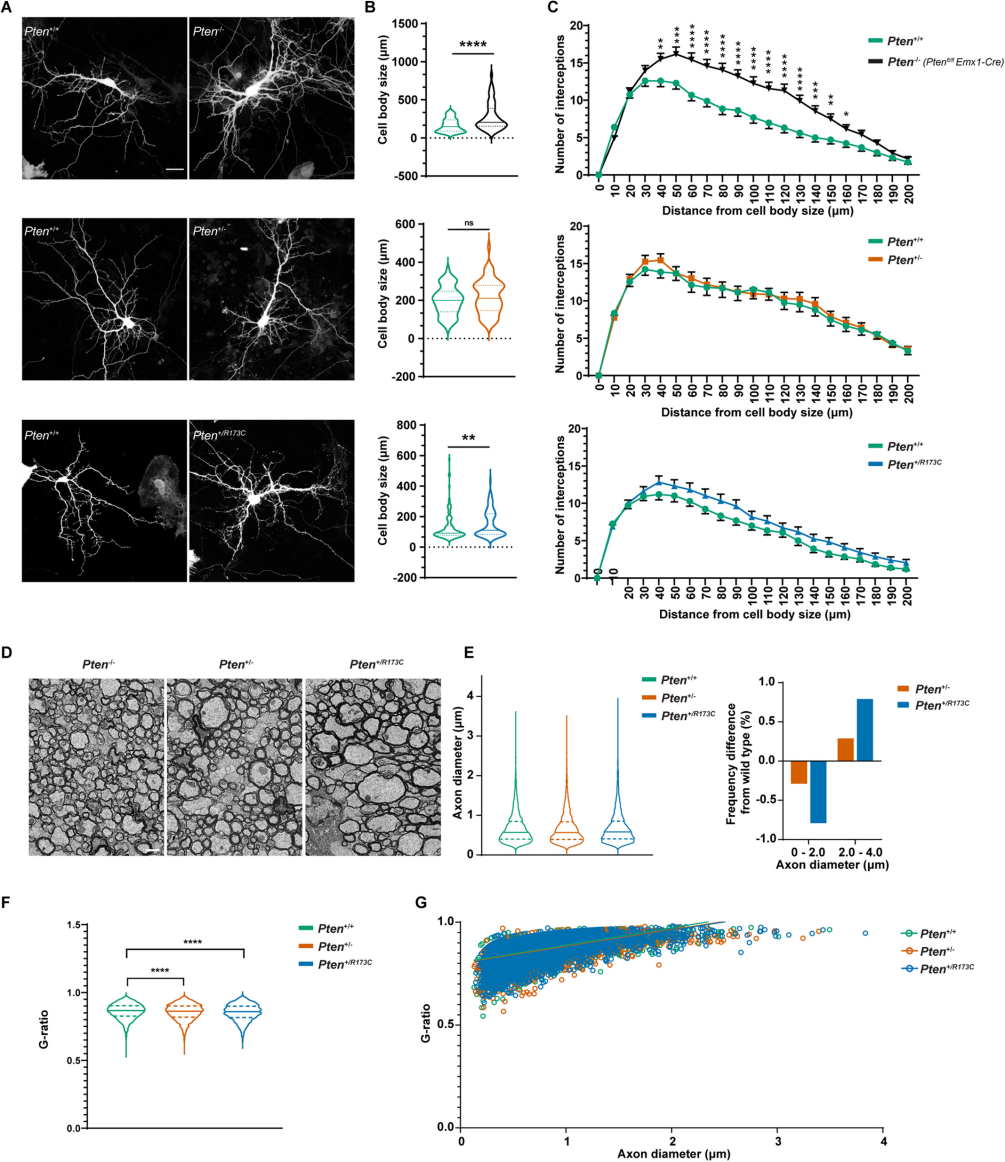
**Neuronal and glial deficits in *Pten^+/R173C^* and *Pten*^+/−^ mice.** (A) Representative images from GFP-transfected cortico-hippocampal neurons at day *in vitro* (DIV)12/13. Neurons were stained for GFP. Scale bar: 20 µm. (B) Quantification of cell body size of neurons shown in A from *n*>60 cells for each genotype from three to six independent experiments. Violin plots show median (thick line) and interquartile range (dotted lines), analysed using Mann–Whitney test. (C) Sholl analysis of neurons shown in A from *n*=63-122 cells from three to five independent experiments shown as mean±s.e.m., analysed using two-way RM ANOVA followed by uncorrected Fisher's LSD test. (D) Representative electron micrographs of the corpus callosum in cross-sections from 3-month-old mice. Scale bar: 1 µm. (E) Quantification of axonal diameters and their frequency difference from >4500 axons measured from *n*=3 mice per genotype. Violin plots show median (thick line) and interquartile range (dotted lines), analysed using Kruksal–Wallis test. (F) Quantification of G-ratios for corpus callosum axons from >4500 axons measured from *n*=3 mice per genotype, analysed using Kruskal–Wallis test followed by Dunn's multiple comparisons test. (G) Scatter plot of G-ratios as a function of axon diameter from >4500 axons measured from *n*=3 mice per genotype. Linear regression analysis showed no statistical differences among the three genotypes. *P<0.05; **P<0.01; ***P<0.001; ****P<0.0001; ns, not-significant (*P*>0.05). Mice used in A-C were on a C57BL/6J background, and those in D-G were on a mixed C57BL/6J×Sv129 background.

To gain further insight into the causes of macrocephaly, we quantified neuronal and glial parameters *in vitro*. Morphologies of cortical pyramidal neurons *in vitro* were assessed following transfection with a GFP expression vector to allow visualisation of the cells (Fig. 5A). *Pten^+/R173C^* neurons had enlarged cell body size and a tendency for increased process complexity (not statistically significant) compared to *Pten^+/+^* controls (Fig. 5B,C). Although cKO neurons demonstrated a more severe phenotype, comparable to that of *Pten^m3m4/m3m4^* neurons with regards to cell body size (Table 2), this phenotype was not observed in *Pten*^+/−^ neurons (Fig. 5B,C) or *Pten^+/m3m4^* neurons (Table 2).

To determine whether glial and/or axonal defects contribute to the enlargement of the white matter, we examined the corpus callosum of *Pten*^+/+^, *Pten^+/R173C^* and *Pten*^+/−^ mice using transmission electron microscopy (Fig. 5D). Although there was no statistically significant difference in the average axonal diameter in *Pten^+/R173C^* mice compared to that in controls, there was a reduction in the number of small diameter axons and a gain of larger diameter axons (Fig. 5E). This phenotype was much less pronounced in *Pten^+/−^* mice (Fig. 5E). Quantification of the G-ratio in the corpus callosum showed an increase in average myelin thickness in both *Pten^+/R173C^* and *Pten*^+/−^ mice (Fig. 5F). However, scatter plot analysis of the G-ratio as a function of axonal diameter did not show significant changes in *Pten^+/R173C^* and *Pten*^+/−^ mice compared to controls (Fig. 5G).

Altogether, our experiments show that the macrocephalic *Pten^+/R173C^* phenotype is caused by alterations in multiple parameters, including increases in neuronal cell body size, axonal thickness, oligodendrocyte lineage cell numbers and myelin thickness.

*Pten^+/R173C^* mice have a similar degree of macrocephaly as *Pten^+/m3m4^* heterozygous mice and have multiple neuronal phenotypes, partially overlapping with those of *Pten^m3m4/m3m4^* and *Pten^+/−^* mice (Table 2). Importantly, the apparently intact or even increased PI3K/AKT regulatory capacity of PTEN-R173C, and the phenotypic overlap with *Pten^m3m4^* mice, suggests that the impaired nuclear function of PTEN is key to the development of macrocephaly and several neuronal phenotypes.

Defective dsDNA damage repair is increasingly being associated with macrocephaly and ASD, with studies showing increased dsDNA breaks in cells isolated from these patients (Wang et al., 2020; Attia et al., 2020). To explore the possible involvement of impaired dsDNA damage repair in neurons in the macrocephalic phenotype, we treated *Pten^+/−^* and *Pten^+/R173C^* mice with IR and examined the levels of γH2AX in the brain 5 h post-IR treatment. Although there was an increase in the γH2AX levels in protein extracts from the brains of both *Pten^+/−^* and *Pten^+/R173C^* mice, compared to those in brain extracts from littermate WT mice, the difference observed was not statistically significant (Fig. S15). This might be due to the IR dosage and/or time of analysis and warrants further investigation.

### *Pten*^+/R173C^ mice display mild behavioural aberrations

ASD is common in PHTS (Yehia et al., 2020). Behavioural abnormalities are found in mice with germline expression of heterozygous loss of function of *Pten* (Clipperton-Allen and Page, 2014) or homozygous expression of the nuclear-excluded *Pten^m3m4^* mutations (Tilot et al., 2014). To examine this in *Pten^+/R173C^* mice, we used a range of tests associated with ASD-like mouse behaviours.

In Crawley's sociability test, *Pten^+/R173C^* mice were indistinguishable from *Pten*^+/+^ littermate controls, indicating normal social interactions (Fig. S16A-D). In the open field test, male mice were not significantly different from their *Pten*^+/+^ littermates with regards to distance travelled and time spent moving (Fig. S17B,C). However, female *Pten^+/R173C^* mice travelled less and spent less time moving than controls (Fig. S17F,G), suggesting decreased exploratory behaviour and/or locomotion. This was not caused by increased anxiety because female (and male) mice spent equal length of time exploring the different compartments of the open field test as controls (Fig. S17D,H), with defecation during the test being comparable between the two groups (Fig. S17E,I). Reduced exploratory locomotion in females was also observed in a hole board test (Fig. S18A). Changes in repetitive behaviours, as assayed in a marble burying test, were absent in both males and females (Fig. S18B).

### Patients with PHTS with nuclear-excluded *PTEN* variants develop malignant cancers later in life

To understand the impact of *PTEN* variants such as *PTEN-R173C* on PHTS pathogenesis, we collated patient phenotype information from published sources, from the PHTS Patient Registry UK, and from personal communication with physicians who see patients with PHTS. These have been summarised in Table 1, with details in Tables S1 and S2. Most patients received PHTS diagnosis at a young age owing to presentation of phenotypes such as macrocephaly (53/59), DD (34/59) and cutaneous pathology (28/59) seen at a high frequency. Patients developed various benign lesions with a moderate frequency such as thyroid cysts or adenomas (10/59) and GI polyps (9/59). Other benign lesions such as endometrial polyps and fibroids, ovarian cysts and lipomas were seen at low frequencies. Vascular malformations, including haemangiomas, venous malformations and arterio-venous malformations, were also seen in adult patients (8/59).

Malignant cancer was seen in few patients with onset later in life. With the exception of one 29-year-old male patient with *PTEN-D310G* variant who developed thyroid and testicular cancer, most patients were above the age of 50. This included two female patients with *PTEN-R173C*; one 63-year-old patient who had developed malignant cancers of the breast, skin and ovary; and a 59-year-old patient with malignant cancers of breast and thyroid. A 54-year-old female patient with *PTEN-R173H* variant developed malignant breast cancer.

The clinical observations in patients with PHTS with *PTEN-R173* variants mirror the phenotypes of *Pten^+/R173C^* mice; although most patients display neurodevelopmental phenotypes early on in life, they develop malignant cancers at a low frequency.

## DISCUSSION

Since the discovery of *PTEN* as a tumour suppressor in somatic cancer, multiple genetic mouse models targeting *Pten* have been generated, highlighting the importance of PIP_3_ phosphatase activity of PTEN in tumour suppression, embryonic development, immunomodulation, neuronal development and the development of autism spectrum traits (Lee and Pandolfi, 2020).

Mouse models have also improved our understanding of PHTS, a rare genetic condition caused by heterozygous germline *PTEN* variants. These include *Pten^+/−^* mice, which represent the heterozygous genetic constellation of *PTEN* in PHTS and on a mixed C57BL6xSv129 background develop multiple phenotypes overlapping with the clinical presentation of PHTS, including brain and lymphoid overgrowth and the development of specific cancers (Tibarewal et al., 2022). Although the PIP_3_ phosphatase activity of PTEN has been the focus of research over the past three decades, there is an increasing body of evidence for other PTEN functions in disease pathogenesis, in particular its nuclear function (Langdon, 2023). Recently, the *Pten^m3m4^* mouse model with germline expression of engineered, nuclear-excluded non-naturally occurring *Pten* mutations, has indicated a role for nuclear PTEN defects in driving neuronal and immune phenotypes in PHTS (Tilot et al., 2014; Jaini et al., 2020).

In this study, we developed a new mouse model featuring the pathogenic *PTEN-R173C* variant commonly found in PHTS and somatic cancer. We report that this *PTEN* variant retains its ability to regulate PI3K/AKT signalling but is impaired in dsDNA damage repair through HR, likely owing to nuclear exclusion. Contrary to previous reports (Han et al., 2000; Smith and Briggs, 2016; Post et al., 2020), we found that PTEN-R173 mutant proteins (R173C and R173H) were catalytically more active PIP_3_ lipid phosphatases than PTEN-WT, both in cells and tissues. However, these mutants displayed reduced protein stability, possibly resulting from a more open protein configuration that is more susceptible to ubiquitination-mediated degradation. Upon heterozygous germline expression in *Pten^+/R173C^* mice, this biochemical feature results in PTEN protein expression levels comparable to those in *Pten*^+/−^ mice but an overall lipid phosphatase activity similar to that of WT mice, as demonstrated by effective downregulation of insulin-stimulated PI3K/AKT signalling.

To assess the importance of nuclear functions of PTEN in tumorigenesis, we aged *Pten^+/R173C^* mice and found only a modest predisposition to solid tumour development compared to that in *Pten*^+/−^ mice. This is reminiscent of mouse models of genes involved in dsDNA damage repair via HR such as *Brca*, *Rad51* and *Palb* (Matos-Rodrigues et al., 2021; Tsuzuki et al., 1996; Roy et al., 2011; Rantakari et al., 2010; Yan et al., 2004; Hakem et al., 1996)*.* Indeed, although homozygous inactivation of these genes frequently leads to embryonic lethality, as also observed in *Pten^R173C/R173C^* mice, their heterozygous inactivation only leads to a very low to absent tumour burden, most likely because the lifespan of mice is insufficiently long to accumulate the additional mutations required for tumorigenesis (Drost and Jonkers, 2009). That additional genetic defects may be required to allow phenotypic output of *PTEN-R173* defects is indicated by the observation that somatic cancers with *PTEN-R173* variants have a range of co-occurring variants, including, but not limited to, oncogenic variants of *PIK3CA* or loss of second allele of *PTEN* (Fig. 3D). Also, mouse models carrying a combination of *Pten* mutations with other oncogenic drivers such as mutant *Pik3ca* and *Erbb2* have accelerated development of mammary tumours (Kato et al., 2020; Yip et al., 2020). Combining the *Pten^R173C^* mutation in mouse models carrying additional cancer drivers (such as *PIK3CA* mutation) or defects in DNA repair genes (such as *Brca* or *Rad51*) could provide evidence for this speculation, but such experiments are outside the scope of the current study.

Our data further show that, in a germline configuration, deficient nuclear localisation of PTEN, and consequently its nuclear function, is associated with brain and lymphoid overgrowth. The role of nuclear PTEN in regulating these phenotypes has been reported, but the mechanism by which nuclear PTEN regulates these phenotypes is unclear. *Pten^+/m3m4^* mice expressing a nuclear-excluded *Pten* mutant develop macrocephaly and lymphadenopathy (Jaini et al., 2020; Mester et al., 2011; Tilot et al., 2014). Increased neuronal cell body size has also been reported in neurons expressing other nuclear-excluded *PTEN* variants such as *PTEN-F241S*, *D252G*, *N276S* and *W274L* (Fricano-Kugler et al., 2018)*.* On the contrary, mice expressing nuclear-excluded, non-pathogenic, *Pten* mutations such as *Pten^K13R/D384V^* have smaller neurons and develop microcephaly (Kato et al., 2021; Igarashi et al., 2018). The discrepancy between these studies may result from mutation-specific changes in PTEN function.

Although our study does not establish a direct link between these overgrowth phenotypes in our mice and dsDNA damage repair, it is possible that such a link exists. Indeed, one study reported increased IR-induced DNA strand breaks in lymphocytes from children with autism compared to those in lymphocytes from unaffected controls, supporting such a functional link (Attia et al., 2020). However, lymphoid cells isolated from patients with PHTS with *PTEN-R173C* variant, who all present with ASD, did not exhibit increased DNA damage following IR treatment (Wei et al., 2024). This suggests that nuclear functions of PTEN other than dsDNA repair underlie the observed lymphoid overgrowth and macrocephaly in our *Pten^+/R173C^* mice. This is further supported by the absence of lymphoid overgrowth and macrocephaly in patients and mouse models with germline mutations in other genes regulating dsDNA damage repair by HR, such as the *Brca*, *Rad51* and *Palb2* genes.

We find that the macrocephalic *Pten^+/R173C^* phenotype is associated with alterations in multiple parameters, including increases in neuronal cell body size, axonal thickness, oligodendrocyte lineage cell numbers and myelin thickness. Although these brain alterations in *Pten^+/R173C^* mice are very clear, their impact on animal behaviour is less prominent. It has to be said that although mice with heterozygous germline expression of *Pten* accurately reflect the genetic makeup of patients with PHTS, the behavioural abnormalities observed in heterozygous mutant mice are often subtle and highly variable (Clipperton-Allen and Page, 2014). This necessitates the use of large mouse numbers to achieve quantifiable significant differences between genotypes. Consequently, the field of behavioural analysis has mostly relied on the use of homozygous mouse models. This approach is particularly true for PHTS models, with numerous studies conducted on homozygous brain-specific *Pten* knockout models (Kwon et al., 2006; Zhou et al., 2009) and homozygous *Pten^m3m4^* models (Tilot et al., 2014). Similar homozygous models are also utilised in behavioural research on other rare diseases such as tuberous sclerosis (Yuan et al., 2012; Bateup et al., 2013). Although these models do not share the exact genetic composition of patients, they serve as platforms for behavioural and neurological studies and testing therapeutics.

Our study suggests that the status of nuclear function of PTEN alongside its PIP_3_ phosphatase activity, allows refinement of genotype/phenotype analysis in PHTS. Based on our findings and assessment of patient phenotypes (Table 1; Tables S1 and S2), our data indicate that individuals carrying *PTEN* variants such as *PTEN-R173C*, which retain their PIP_3_ phosphatase function but are nuclear excluded, tend to follow a characteristic progression of known PHTS disease phenotypes: early signs such as neurological abnormalities – including macrocephaly and global developmental delay – alongside cutaneous features, which together lead to a PHTS diagnosis during childhood. As they enter early adulthood, they may be prone to developing vascular abnormalities and benign tumours in the thyroid and GI tract, with an increased likelihood of developing malignant cancers later in life.

Our study also highlights the possibility of new therapeutic treatments for cancer in patients with PHTS. A previous study has shown that glioma cells expressing PTEN-Y204F, a mutation that leads to lack of PTEN nuclear localisation and functions such as dsDNA damage repair, are sensitive to radiation therapy (Ma et al., 2019). It is therefore tempting to speculate that radiation therapy and dsDNA damage repair antagonist drugs, such as *PARP* inhibitors, might be additional therapeutic options beyond PI3K pathway inhibitors for cancer in patients with PHTS carrying *PTEN-R173*-like variants.

## MATERIALS AND METHODS

### Plasmids

DNA constructs are detailed in Table S8. Point mutations were introduced in PTEN WT complementary DNA (cDNA) in different vectors using the primers listed in Table S9 and KOD hot start DNA polymerase (Sigma-Aldrich) according to the manufacturer's instructions.

### Mice

All mice were maintained at University College London (UCL) in accordance with the UK Animals (Scientific Procedures) Act 1986 and following UK Home Office guidance. All procedures were authorised by a UK Home Office Project Licence subject to local ethical review. *Pten*^+/−^ mice [*Pten^tm1Rps^*; MGI:2151804 (Podsypanina et al., 1999)], *Pten*^flox/flox^ mice [*Pten^tm1Hwu^*; MGI:2156086 (Lesche et al., 2002)], *CAG CreER^T2^* mice [*Gt(ROSA)26Sor^tm3(CAG−Cre/ERT2)Dsa^*; MGI:5616874 (Schonhuber et al., 2014)] and *Emx1-Cre* mice [*Tg(Emx1-cre)1Wdr*; MGI ID:3761167 (Kessaris et al., 2006)] have been described elsewhere. *Pten^+/R173C^* mice were generated by Taconic Biosciences using a CRISPR/Cas9 approach. Guide RNA (gRNA) was designed to contain the R173C mutation (c.517C>T) and a silent mutation containing the *Eco*NI restriction site upstream of the R173 site (to allow discrimination of WT and knock-in allele following PCR). Fig. S6 shows the gRNA sequence and the targeting strategy. gRNA was microinjected into C57BL/6J mouse zygotes along with recombinant Cas9. The zygotes were then transferred to pseudo-pregnant mothers. The litters born (founder animals) were sequenced to check for the incorporation of the R173C mutation and percentage of mosaicism. They were then used as a breeder to generate F1 animals. F1 males positive for the R173C mutation and negative for any off-target mutations were used to generate further animals by *in vitro* fertilisation using C57BL/6J females. All mouse lines were maintained on a C57BL/6J background. Sv129 mice were purchased from Charles River Laboratories or Envigo. *Pten*^+/−^ and *Pten^+/R173C^* mice were crossed with Sv129 mice to obtain mixed background F1 mice that were used for some experiments as described. For neuronal studies, conditional knockout mice that selectively lack PTEN expression in cortical pyramidal neurons were generated by crossing *Pten^flox/flox^* mice with *Emx1-Cre* mice to obtain *Emx1-Cre*;*Pten^fl/fl^*, referred to here as cKO, mice. The cKO mice were crossed with *Pten^+/R173C^* mice to obtain *Emx1-Cre*;*Pten^fl/R173C^*, referred to here as cKI, mice, which express one only copy of *Pten*-R173C in the cortical pyramidal neurons.

For genotyping, mouse tissue samples (from ear biopsy or from the embryonic yolk sac) were lysed in 25 mM NaOH/0.2 mM disodium EDTA for 1 h at 95°C. The samples were neutralised by adding an equal volume of a buffer containing 40 mM Tris-HCl, pH 4.5. For PCR using a titanium polymerase PCR kit (Takara) and the primers listed in Table S10, 2 µl of the DNA extract was used. For genotyping of *Pten^+/R173C^* mice, an additional restriction digestion step was performed on the PCR products using *Eco*NI (New England Biolabs).

### Mouse survival studies

For sample size calculations for long-term survival study, we use ClinCalc. The mean survival age of *Pten*^+/−^ on a mixed C57BL6J×Sv129 background has been reported to be 12±1.8 months (Alimonti et al., 2010). The sample size required for *Pten^+/R173C^* mice needed to see a 15% improvement in mean survival age compared to that of *Pten*^+/−^ mice was calculated as 16 (probability of type-I error was set to 0.05 and power was set to 80%). We used at least 16 mice for each genotype and each sex. At least ten littermate WT mice were used for both *Pten*^+/−^ and *Pten^+/R173C^* mice. For survival analysis, mice were weighed twice a week. They were monitored and euthanised upon showing a more than 20% reduction in body weight; any signs of ill health such as hunched appearance, laboured breathing and sluggish behaviour; or palpable masses with a total surface area of 1.4 cm^2^ surface area. Animals showing no clinical signs were euthanised at the end of the study (600 days for females and 730 days for males).

### Cell lines

U-87 MG (U87), HEK-293T and Phoenix cells were purchased from American Type Culture Collection; Lenti-X™ 293T cells were purchased from Clontech. Human PTEN knockout HeLa cell line and control HeLa cells were obtained from Abcam (ab255419 and ab255928, respectively); PTEN knockout was validated using western blotting (Fig. S4C). All cell lines were maintained in Dulbecco's modified Eagle medium (DMEM; Sigma-Aldrich), supplemented with 10% filtered bovine serum (FBS; Pan-Biotech) and penicillin-streptomycin (Pen-Strep; Sigma-Aldrich) using standard cell culture methods. All cell lines were routinely tested for mycoplasma contamination.

### Preparation of MEFs

For preparation of E13.5 MEFs, timed matings were set up between *Pten*^+/−^ mice and *Pten^+/R173C^* mice, and *Pten^flox/+^CreER^T2^* and *Pten*^+/−^ or *Pten^+/R173C^* mice. Pregnant females were euthanised on E13.5, and embryos were harvested from the uterus. Following removal of the head and visceral organs, the rest of the embryo was minced in 2× trypsin/EDTA using a sterile scalpel and incubated at 37°C for 40 min. The cell suspension was plated on 10 cm dishes in DMEM (Sigma-Aldrich) supplemented with 10% FBS (Pan-Biotech) and Pen-Strep (Sigma-Aldrich), and cells were maintained using standard cell culture procedures. For preparation of E9.5 MEFs, timed intercrosses were set up between *Pten^+/R173C^* mice. Pregnant females were euthanised on E9.5, and embryos were removed from the uterus and gently dissociated by pipetting up and down using a p1000 pipette and 2 ml media-containing DMEM (Sigma-Aldrich), supplemented with 10% FBS (Pan-Biotech) and Pen-Strep (Sigma-Aldrich). The cell suspension was plated in 48-well plates, and cells were maintained using standard cell culture procedures. To induce Cre-mediated recombination of the floxed *Pten* allele in *Pten^flox/+^CreER^T2^* MEFs, 4-hydroxy tamoxifen (Sigma-Aldrich) was added to the cells at a final concentration of 1 µM and refreshed every 24 h for 3 days.

### Primary neuron culture

Primary cortico-hippocampal cultures were prepared from postnatal day (P)1 or P2 pups after genotyping. Cortex and hippocampus were dissected, digested with trypsin and dissociated using glass pipettes, and 40,000 cells were plated per 12 mm coverslip. Neurons were cultured in serum-free medium [neurobasal (NBA) medium supplemented with B27 supplement, L-glutamine, penicillin/streptomycin and 10 mM HEPES pH 7.4]. Cells were cultured at 5% CO_2_ at 37°C with replacement of half of the medium every 2-3 days. Cells were transfected on day *in vitro* (DIV)6 with a GFP expression vector using Lipofectamine 3000 according to the manufacturer's instructions. On DIV12/13, cells were fixed with 4% paraformaldehyde (PFA)/4% sucrose in PBS for 10 min at room temperature (RT) and washed 3× in PBS. Cells were blocked and permeabilised in immunofluorescence (IF) buffer [PBS supplemented with 0.3% Triton X-100 and 3% bovine serum albumin (BSA)] for 20 min and incubated with primary antibodies against NeuN (also known as RBFOX3; Millipore MAB377, 1:1000) and GFP (Abcam, ab290, 1:6000) for 2 h at RT in IF buffer in a humidity chamber. After three washes with PBS, cells were incubated with cross-absorbed Alexa Fluor-labelled secondary antibodies (Invitrogen) in IF buffer for 1 h at RT in a humidity chamber in the dark, washed 3× with PBS and mounted with Fluoromount-G (Invitrogen). Neurons were imaged using a LSM 880 microscope (Leica) acquiring *z*-stacks at 40× magnification. Dendritic complexity was determined using the Sholl macro in FIJI. Interceptions were analysed in 10 µm radii within a 200 µm distance from the centre of the soma. To measure cell body size in FIJI, stacks were processed to *z*-projections, regions of interest (ROIs) were drawn manually around the cell bodies, and the ROI area was measured in FIJI.

### PTEN activity assays

For purification of bacterially expressed GST-tagged PTEN proteins, pGEX6P1 vectors with cDNA for WT or mutant PTEN were introduced in *E. coli*, and recombinant PTEN was purified by glutathione-affinity chromatography followed by cleavage of the GST tag as described (Davidson et al., 2010). PIP_3_ assays using purified recombinant PTEN were performed with diC8 PIP_3_ (Cell Signals Inc.) and a malachite green phosphate assay kit (Sigma-Aldrich) and have been described elsewhere (Maehama and Dixon, 1998). Purification of recombinant PTEN protein from insect cells was as described in Masson et al. (2016b).

### Lentiviruses and retroviruses

Lentiviruses for PTEN and PIP_3_ biosensor were generated by co-transfecting Lenti-X™ 293T cells or HEK-293T cells with plasmids expressing the cDNA of interest along with packaging vectors using TransIT-LT1 following the manufacturer's instructions. For preparation of retroviruses, Phoenix cells were transfected with p53 short hairpin RNA (shRNA)-expressing vector (Dickins et al., 2005) using TransIT-LT1 following the manufacturer's protocol. 24 h post transfection, sodium butyrate was added to the cells to a final concentration of 12.5 mM for 6 h, followed by washing the cells with warm PBS and addition of fresh medium. The supernatant containing lentiviral particles was collected after 20 h, passed through a 0.45 µm filter and stored at −80°C. Target cells for transductions were plated at 40-50% confluency. Once the cells had attached, lentiviral/retroviral particles were added to the cells along with polybrene (Sigma-Aldrich) at 20 µg/µl. The medium was changed 24 h post transduction.

### Immunoblotting analysis

Protein extracts from cell cultures were prepared by scraping cells into lysis buffer containing 25 mM Tris-HCl pH 7.4, 150 mM NaCl, 1% Triton X-100, 10% glycerol, 1 mM EGTA, 1 mM EDTA, 5 mM sodium pyrophosphate, 10 mM β-glycerophosphate, 50 mM sodium fluoride, 1 mM sodium orthovanadate, 1 mM DTT and protease inhibitor cocktail (Millipore). Mouse tissues harvested from mice upon euthanasia were homogenised using Lysing Matrix M tubes (MP Biomedicals) in double the volume (v/w) of lysis buffer (25 mM Tris-HCl pH 7.4, 150 mM NaCl, 1% Triton X-100, 0.1% SDS, 10% glycerol, 1 mM EGTA, 1 mM EDTA, 10 mM sodium pyrophosphate, 20 mM β-glycerophosphate, 100 mM sodium fluoride, 2 mM sodium orthovanadate, 1 mM DTT and protease inhibitors) on a FastPrep 24 homogeniser (MP Biomedicals) at 4 m/s for 20 s. Cell or tissue lysates were pre-cleared by centrifugation at 20,000 ***g*** for 10 min, and protein extracts were analysed by immunoblotting. HEK-293T cells expressing Rluc-PTEN-YFP constructs were lysed in buffer containing 50 mM HEPES (pH 7.4), 250 mM NaCl, 2 mM EDTA, 0.5% NP-40, 10% glycerol supplemented with protease and phosphatase inhibitors (Roche) and protein A/G PLUS-Agarose (30 µl/sample), and mouse anti-GFP antibody (Roche, 11814460001, 1:200) was used to immunoprecipitate YFP-tagged PTEN protein, which was used for immunoblotting as described below. Protein gel electrophoresis was conducted with 10 µg total soluble protein per lane on NuPage Bis-Tris 4-12% gradient polyacrylamide gels (Thermo Fisher Scientific) following the manufacturer's protocols. Proteins were transferred onto PVDF membrane (Millipore), and membranes were blocked in 5% milk powder/Tris-buffered saline with Tween-20 (TBST) for 1 h at RT. Blocked membranes were incubated overnight with primary antibodies (Table S11). Antibody complexes were detected by 1 h incubation at RT with horseradish peroxidase (HRP)-conjugated secondary antibodies (GE Healthcare). Blots were developed with Immobilon Forte Western HRP substrate (Millipore) and chemiluminescence imaged using an ImageQuant LAS4000 imaging system (GE Healthcare).

### Quantitative RT-PCR assays

Total RNA was extracted from U87cells non-transduced or transduced with lentiviruses for PTEN-WT, PTEN-C124S, PTEN-R173C or PTEN-R173H, using an RNAEasy kit (Qiagen) following the manufacturer's instructions. 1 µg RNA was converted to cDNA using an iScript RT kit (Bio-Rad). 50 ng cDNA was used for quantitative RT-PCR reactions using the primers in Table S12. The reactions were run on a QuantStudio™ 5 Real-Time PCR machine (Applied Biosystems). dCT was calculated by deducting the CT for GAPDH from the CT for PTEN. ddCT was calculated by deducting the dCT of untransduced cells from the dCT of the sample of interest.

### Cycloheximide chase studies

U87 cells were transduced with lentiviruses for PTEN-WT, PTEN-C124S, PTEN-R173C or PTEN-D252G. 48 h post transduction, cells were treated with 200 µg/ml cycloheximide and lysed at different time points, and protein extracts were analysed for PTEN expression by immunoblotting.

### BRET assays

BRET assays using the Rluc-PTEN-YFP biosensor were performed as described previously (Misticone et al., 2016; Lima-Fernandes et al., 2014). Briefly, 24 h post transfection, cells were detached from 12-well plates, using trypsin-EDTA, and resuspended in 1 ml complete medium. The cells were subsequently distributed into poly-L-ornithine-coated (30 µg/ml) white 96-well optiplates (Perkin Elmer). The next day, medium was replaced with Opti-MEM (Gibco), and coelenterazine substrate (Interchim) was added to a final concentration of 5 µM and incubated for 3 min at 25°C. BRET readings were then collected using a Multilabel Reader Mithras2 LB 943 (Berthold Technologies). Sequential integration of light output was measured for 1 s using two filter settings (480+10 nm for YFP and 540+20 nm for Rluc). The BRET signal represents the ratio of the light emitted by YFP and the light emitted by Rluc (YFP/Rluc). The ratio values were corrected by subtracting background BRET signals obtained with Rluc-PTEN. MilliBRET (mBRET) values were calculated by multiplying these ratios by 1000. Data are represented as specific mBRET values or change in mBRET (ΔmBRET) when compared to the signal obtained with WT PTEN set to 0.

### MTS assays

U87 cells were transduced with lentiviruses for GFP, PTEN-WT, PTEN-C124S, PTEN-R173C or PTEN-R173H. 48 h post transduction, 5000 cells/well were plated in 96-well plates. A different plate was prepared for each day. 20 µl MTS assay reagent (Abcam) was added to the cells, which were then incubated at 37°C for 1 h, followed by measurement of absorbance at 490 nm using a plate reader.

### PIP_3_ biosensor analysis and PTEN localisation using fluorescence microscopy

For plasma membrane PIP_3_ quantification, U87-MG cells transduced with lentiviral particles expressing the PIP_3_ biosensor on its own or with lentiviral particles for PTEN-WT or PTEN-R173C, were pretreated with or without GDC-0941 (1 µM) for 1 h and stimulated with or without insulin (100 nM) for 2 min. For PTEN localization studies, MEFs were treated with or without GDC-0941 (1 µM) for 1 h prior to fixation. For both plasma membrane PIP_3_ quantification and localisation of untagged or endogenous PTEN, cells were fixed with 4% PFA for 20 min, washed in PBS, permeabilised with 0.1% Triton X-100 in PBS for 90 s, washed three times with PBS and blocked with PBS containing 1 or 3% (w/v) BSA for 30 min. Primary antibodies against PTEN were diluted in 1% or 3% BSA block solution and incubated for 1 h at RT. Following three washes with PBS, secondary antibodies and Phalloidin were diluted in 1% or 3% BSA block solution and incubated for 45 min at RT. Cells were then washed three times and mounted using ProLong Gold antifade reagent containing DAPI (Life Technologies, P36935). To examine GFP-PTEN localisation during different stages of the cell cycle, Lenti-X™ 293T cells were transfected with DNA for PTEN-WT-GFP or PTEN-R173C-GFP. 18 and 36 h post transfection, the cells were subjected to double thymidine block as below. The same procedure was used for *Pten^−/R173C^* and *Pten^R173C/R173C^* MEFs. For double thymidine block, the culture medium was changed to fresh medium containing 2 mM thymidine (Sigma-Aldrich). 18 h post the second medium change, the cells were released from double thymidine block by changing into fresh, complete medium. Cells were fixed with 4% PFA for 15 min, washed three times in PBS and permeabilised with 0.1% Triton X-100 in PBS for 5 min. Cells were then washed three times with PBS and once with water before mounting using ProLong Gold antifade reagent containing DAPI. Confocal microscopy was performed using a Zeiss LSM 880 microscope with AiryScan and a 63× PL APO objective and Zen Black acquisition software or Zeiss LSM900 microscope and a 63× or 40× PL APO objective and Zen Black acquisition software. Image processing was performed using ImageJ software (National Institutes of Health) and was limited to alterations of brightness, subjected to the entire image. Quantification of plasma membrane PIP_3_ intensity was performed using ImageJ as described previously with minor modifications (Ooms et al., 2015). The average fluorescence intensity was measured within a box of defined size (20×10 pixels) at three random regions of the plasma membrane and three random regions of the cytosol (box size 20×20 pixels). The plasma membrane and cytosol fluorescence intensity measurements were each averaged, and the ratio of plasma membrane to cytosolic fluorescence intensity was determined. The PTEN nuclear to cytoplasmic ratio was measured using ImageJ. First, the nuclear PTEN fluorescence intensity was measured, followed by the cytoplasmic PTEN fluorescence intensity. The ratio of nuclear to cytoplasmic PTEN fluorescence intensity was then calculated.

### Insulin tolerance test (ITT)

3-month-old male *Pten*^+/−^ and *Pten^+/R173C^* mice and their WT littermates were starved for 6 h. The tip of the tail was punctured using a 26 G needle, and time-0 blood glucose levels were recorded using a standard glucometer. The mice were given an intraperitoneal injection of insulin to a final concentration of 0.75 U/kg. Blood glucose readings were recorded at 15 min, 30 min, 45 min, 60 min, 90 min and 120 min post injection.

### Irradiation of mice

8- to 12-week-old mice were subjected to whole-body irradiation of 7 Gy dose using the Small Animal Radiation Therapy Platform (SARRP). 5 h post irradiation, the mice were euthanised with a lethal injection of pentobarbital. Tissues of interest were harvested and snap frozen in liquid nitrogen for biochemical analysis.

### Flow cytometry

Mouse spleens and lymph nodes were mashed with a syringe plunger in RPMI containing 10% FBS and filtered through a 70 µm filter. Cell suspensions from spleen were treated with RBC lysis buffer (BioLegend). The cell suspensions were washed with PBS and stained with Fixable Viability Dye eFluor™ 780 (Thermo Fisher Scientific) in PBS for 40 min followed by a few PBS washes. For staining of surface antigens, antibodies were diluted in fluorescence-activated cell sorting (FACS) staining buffer (PBS+2% FBS, 2 mM EDTA) at 1:100 and added to the cells for 30 min, followed by two washes with FACS buffer. Details on antibodies and reagents used are shown in Table S11. The stained cells were analysed on a BD FACSymphony™ analyser.

### Tissue processing for histological analysis and immunohistochemical analysis

Mouse tissues were fixed in 10% neutral buffered formalin for 24 h, followed by processing and embedding into paraffin blocks. The tissues were orientated with the biggest surface down, to obtain complete sections. 3 µm serial sections were cut and stained with Haematoxylin and Eosin, followed by immunohistochemistry staining on the Leica BOND Rxm. Tissue sections for immunohistochemistry were incubated for 40 min at 100°C in BOND epitope retrieval solution 2 (Leica), followed by blocking and staining using a BOND polymer refine detection kit (Leica, DS9800), primary antibody (Table S11) and DAB enhancer (Leica). All tissue slides except brain were analysed by C.L.S., a veterinary pathologist.

### Histopathological analysis

Non-neoplastic lesions, e.g. splenic extramedullary haematopoiesis and lymph node hyperplasia, were graded using a standard semi-quantitative grading scheme from 0 to 5, where 0=lesion not present and 5=the maximum lesion size/extent (Scudamore, 2014). In general, where present, the presence of other proliferative lesions, including hyperplasia and neoplasia, was recorded for each individual organ, allowing the overall incidence to be established for each lesion in each group. PIN was graded as low or high grade according to the consensus suggested by Ittmann et al. (2013). Low-grade PIN lesions are focal, with one to two layers of cells and mild nuclear atypia. High-grade lesions tend to be more extensive, filling the prostatic lumen but without stromal invasion; have two or more cell layers often forming papillary or cribriform patterns; and have increased nuclear atypia and mitoses.

### Perfusion and preparation of brain sections for DAB immunostaining and immunofluorescence

Mice were perfused with saline (0.9% NaCl) followed by 4% PFA (w/v) in PBS through the left ventricle of the heart. Brains were removed and post-fixed overnight at 4°C in 4% PFA, cryoprotected in 20% sucrose in PBS for 24 h at 4°C and embedded in Tissue-Tek OCT compound (Sakura) in isopentane cooled on dry ice. Frozen brains were stored at −80°C until use. Brains were processed on a cryostat (30 µm thickness), and sections were collected in PBS for immunohistochemistry. For DAB immunostaining, floating sections were incubated with 0.3% H_2_O_2_ in PBS for 30 min while shaking at RT, and then blocked using blocking buffer (10% FBS in PBS supplemented with 0.1% Triton X-100) for 1 h at RT. After blocking, sections were incubated overnight at 4°C with primary antibodies diluted in blocking buffer. Primary antibodies were detected with biotinylated secondary antibodies (Donkey Anti-Mouse IgG Biotinylated Antibody, R&D Systems) diluted 1:300 in blocking buffer for 1 h at RT. The signal was amplified using the Vectastain ABC Kit HRP kit (Vector Laboratories, PK-6100), according to the manufacturer's instructions and developed using a DAB Peroxidase (HRP) Substrate Kit (Vector Laboratories, SK-4100) until the desired staining intensity was reached. Floating sections were mounted on SuperFrost Plus slides (Thermo Fisher Scientific) in 0.2% pork skin gelatine dissolved in 50 mM Tris-HCl pH 7.4, dried at RT overnight, and dehydrated in ethanol and xylene before mounting with DPX mounting medium (Merck). Images were acquired at 20× magnification on a Zeiss Axio Scan.Z1 microscope and processed using Zeiss ZEN lite software. For immunofluorescence, sectioned brains were blocked for 1 h before primary antibodies were applied overnight. Alexa Fluor 488 secondary antibody was used to detect OLIG2 (1:1000; Millipore, AB9610) for 60 min at room temperature together with Hoechst 33258 (1:1000; Sigma-Aldrich, ATEH99E40BB0) to detect cell nuclei. All secondary antibodies were diluted in block solution. Floating sections were transferred onto SuperFrost Plus slides (BDH Laboratory Supplies) and air dried before being coverslipped with Dako fluorescent mounting medium.

### Analysis of corpus callosum and cortex thickness and cell counting

Matched sections at specified Bregma levels were measured using the Zeiss Blue software measurement length tool. Counting boxes were positioned onto matched slices in the barrel field of the primary somatosensory cortex perpendicular to the corpus callosum. In each box, cells were counted from the border of the corpus callosum to the pial surface. The length of the box was adjusted to fit the cortical thickness, while the width was not changed. All cells positive for NeuN within the box were counted, and cell numbers displayed as crude numbers in the box as well as density considering the area of the boxes analysed. Four animals per genotype were used, and quantifications were performed on four hemispheres from two consecutive sections.

### Electron microscopy (EM)

EM was performed as described in Kougioumtzidou et al. (2017). Briefly, mice were prepared for EM by PBS perfusion followed by 2.5% (v/v) glutaraldehyde and 2% (w/v) PFA in 0.1 M sodium cacodylate buffer (pH 7.6). Brains were removed and kept at 4°C for 48 h in the same fixative, before being washed with 0.1 M cacodylate buffer, rinsed briefly with 0.1 M phosphate buffer and shipped to Japan at ambient temperature for processing and electron microscopy. Sagitally sectioned brains were processed for EM. After immersion in a 1% (w/v) osmium tetroxide solution for 2 h at 4°C, the specimens were dehydrated through a graded alcohol series and embedded in Epon 812 (TAAB Laboratories). The required area was trimmed, and ultrathin sections were cut, collected on a platinum-coated glass slide, stained with uranyl acetate and lead citrate and imaged in a scanning electron microscope equipped with a back-scattered electron beam detector (Hitachi SU8010) at 1.5 kV accelerating voltage. EM images of sagittal sections of corpus callosum were taken at 15,000× magnification. To estimate G-ratios, the circumference of the axon and the external circumference of the myelin sheath were measured using ImageJ, avoiding paranodal areas. The diameters of axon and myelin sheath were calculated from these circumferential measurements, assuming a circular profile, and the G-ratio was calculated.

### Behavioural analysis

Behaviour testing was carried out as previously described (Magno et al., 2021). 12-week-old mice on mixed C57BL/6×Sv129 background were used in this study. All mice used were group housed (maximum five animals per cage) with standard bedding without environmental enrichment in a room with a 12 h light and 12 h dark cycle and with food and water *ad libitum*. Experiments took place during the 12 h light cycle between 09:00 and 17:00 in a room in which external sounds were masked by white noise. All sessions were video recorded for analysis purposes. All procedures for the care and treatment of animals were in accordance with the Animals (Scientific Procedures) Act 1986. The indicated numbers of WT mice and their *Pten^+/R173C^* littermates were tested in the open field test, three chambers test, hole board test and marble burying test. Mice tested in behavioural experiments were habituated to handling and the experimenter for 9 days before the start of behavioural experiments to reduce stress, and habituated mice were generally handled by cupping. Mice were analysed in three cohorts, and male and female mice were tested on separate days. During experiments, white noise was played at ∼50 Db to mask external noises, and all experiments were video recorded. Before every experiment, mice were brought into the behavioural room 30 min before the first experiment for habituation, and animals that had finished a test were transferred into a new cage in order not to influence the behaviour of the remaining experimental animals. Directly before the first experimental animal was tested, and, after every mouse, the set up (open field, sociability chamber, hole board) was cleaned thoroughly with water and 70% ethanol in a consistent manner for every animal.

#### Open field

The experimental animal was placed in an open field box measuring 30 cm×30 cm. The walls were white and 40 cm high so that the animal could not get any visual clues. The mouse was allowed to roam freely for 30 min to explore the arena without any prior habituation. Distance, time spent in different areas and the time moving were analysed using Ethovision software while faecal boli were counted manually.

#### Sociability

The experimental animal was placed into the central compartment of a Crawley's chamber for 5 min with closed doors for habituation. After 5 min, empty cages were placed into the middle of the left and right compartments, and a stranger mouse was gently put into one of the cages (counterbalance left and right sides). The stranger mouse matched the subject mouse with regards to the breeding background, age and sex and had no previous contact with the subject mouse. Both doors were opened at the same time, and the experimental mouse was allowed to explore the area for 10 min. A stopwatch was used to determine the time the subject mouse spent closely interacting with either cage (direct contact/sniffing/cage climbing). The time spent with the empty cage versus the first stranger was measured, referred to as sociability. Calculation of discrimination indices was as follows: (time spent with mouse−time spent with empty cage)/(sum of the time spent with both).

#### Hole board

As the experimental animals were familiar with the open field arena from the open field experiment, they were only briefly re-exposed to the empty arena for 10 min on day 1 of the hole board test. The next day, a hole board with 16 holes evenly spaced was placed into the arena, and the subject mouse was put into the middle of the field and was recorded for 15 min. A manual cell counter was used to determine the total number of head dips into the holes.

#### Marble burying

A cage was filled with 5 cm bedding that was tamped down lightly to produce an even surface. 12 glass marbles were distributed evenly spaced in the cage, and the animal was allowed to roam freely for 15 min. Marbles that were buried to 2/3 of their depth with bedding were visually counted.

### Statistical analysis

GraphPad Prism was used for statistical analysis. The method used for individual datasets is indicated in the figure legends. Details such as post hoc tests and *P*-values are listed in Table S13.

### Patient data

Patient data in Table 1, Tables S1 and S2 were collected from the PHTS Patient Registry UK, which has patient consent for data release. K.L. and I.A.M. provided data with patient consent. All clinical investigations were conducted according to the principles expressed in the Declaration of Helsinki. The remaining data are from published sources (see Tables S1 and S2).

### Generation of oncoplot

Samples with *PTEN-R173* variants (*n*=21) were identified across cBioPortal PanCancer datasets (accessed February 2020). All somatic mutations across all genes were extracted for these 21 samples to identify co-occurring genetic alterations. An oncoplot was created using the maftools R package, which displays the top 20 most frequently mutated genes among *PTEN-R173* samples.

## Supplementary Material

10.1242/dmm.052527_sup1 Supplementary information

Table S1. Clinical findings of PHTS patients with nuclear excluded but catalytically active PTEN variants

Table S2. Clinical findings of PHTS patients with PTEN-R173 mutations

Table S3. Reason for euthanasia of Pten^+/+^, Pten^+/-^ and Pten^+/R173C^ mice

Table S4. Frequency and grade of lesions in male mice

Table S5. Frequency and grade of lesions in female mice

Table S6. Frequency and grade of genetic background specific and immune findings in male mice

Table S7. Frequency and grade of immune findings in female mice

Table S8. DNA constructs

Table S9. Primers for site-directed mutagenesis

Table S10. Primers used for genotyping

Table S11. Antibodies used

Table S12. qRT PCR primers

Table S13. Statistical tests and p values
