# Supplementary material for: Impaired nuclear PTEN function drives macrocephaly, lymphadenopathy and late-onset cancer in PTEN hamartoma tumour syndrome
Source: Dis Model Mech. 2026 Jan 7;19(1):dmm052527. doi: 10.1242/dmm.052527 (PMC12817337; doi:10.1242/dmm.052527)
Supplement: Supplementary information [file dmm-19-052527-s1.pdf]

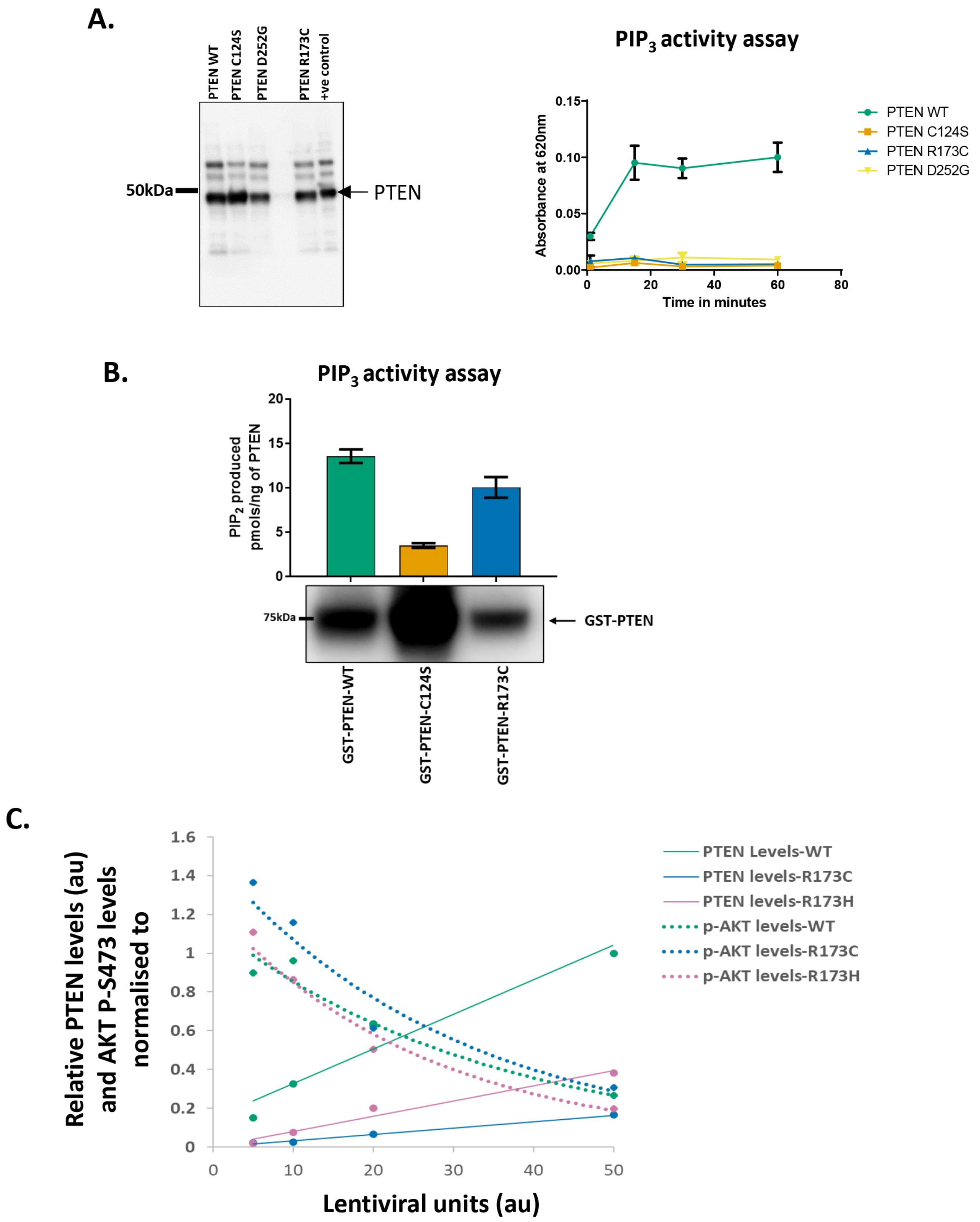

Disease Models & Mechanisms • Supplementary information

Fig. S1. Characterisation of PTEN-R173C. (**A**) Recombinant untagged PTEN wild-type (WT) and mutant proteins were purified from *E. coli* and used in a PIP_3 lipid phosphatase assay. *Left*, Coomassie blue-stained SDS-PAGE gel showing the level of purified recombinant protein. A previous batch of purified PTEN protein is used as a positive control. *Right,* Graph showing PTEN activity measured as absorbance at 620 nm in a malachite green assay. Data shown as mean ±SEM from n=3. (**B**) Affinity-purified GST-tagged PTEN wild-type and mutant proteins expressed in Lenti-X 293T cells were subjected to a PIP_3 lipid phosphatase assay. Graph shows PIP_2 produced per ng of recombinant PTEN from one experiment. Data show mean±SEM from three technical replicates. Immunoblot of GST-PTEN expression levels is shown under the graph. (**C**) Quantification of PTEN and p-AKT from blots from Fig. 1B, au: arbitrary units. p-AKT levels have been normalised to untransduced controls.

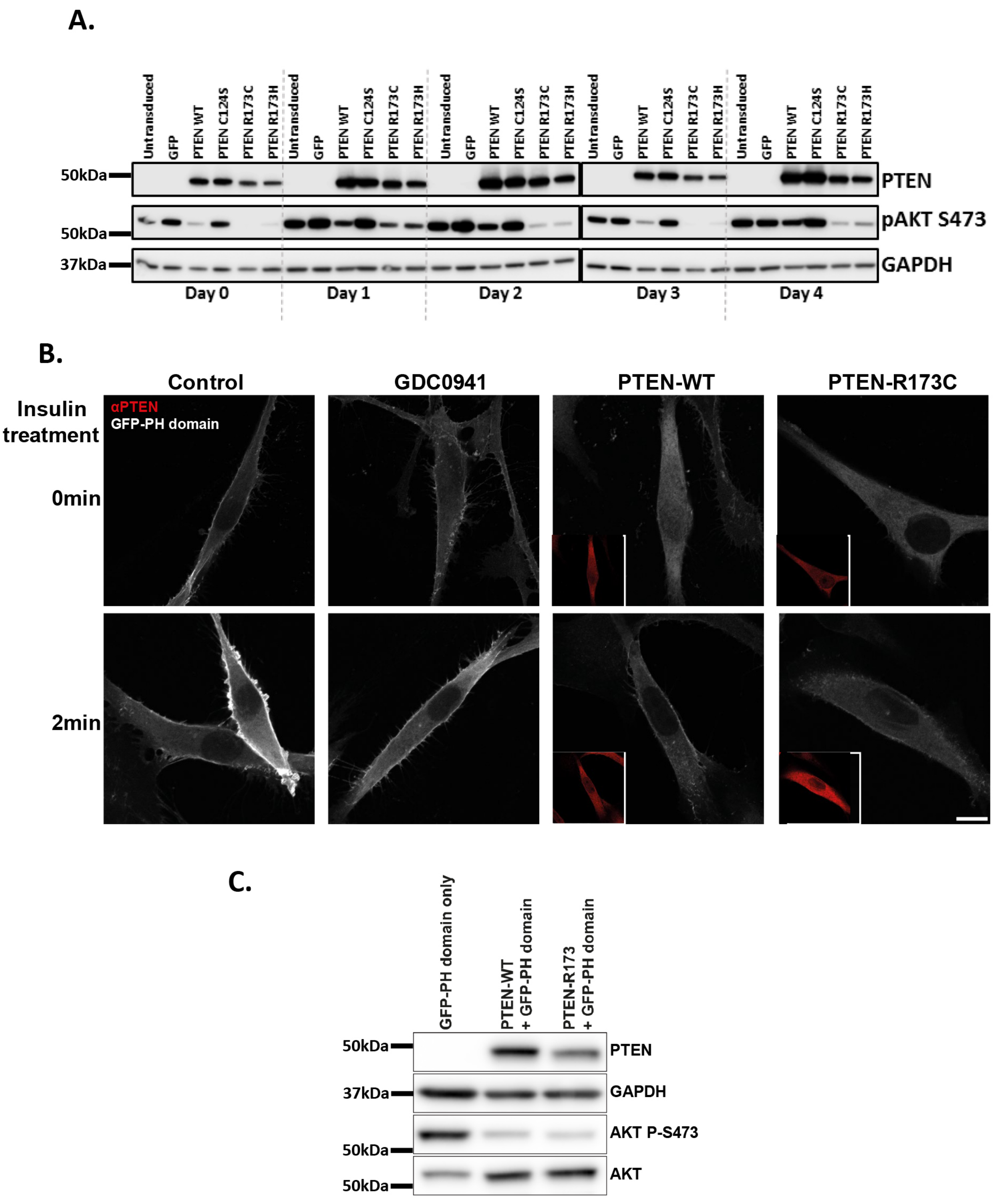

Fig. S2. Characterisation of PTEN-R173C. (A) Immunoblots of PTEN protein and pAKT-473 from the cells in Fig. 1C. **(B)** U87 cells, transiently transduced with lentivirus encoding a PIP_3 biosensor, with or without PTEN-WT or PTEN-R173C, were pre-treated + GDC-0941 (1 µM) for 1 h, followed + insulin treatment (100 nM) for 2 min. The cells were stained with antibodies to PTEN (red inset) and imaged by confocal microscopy. Quantification of the mean fluorescence intensity (MFI) of the PIP3 biosensor is shown in Fig. 1D. (C) Immunoblots of PTEN, AKT P-S473, AKT and GAPDH from the cells in (B) and Fig. 1D.

Disease Models & Mechanisms • Supplementary information

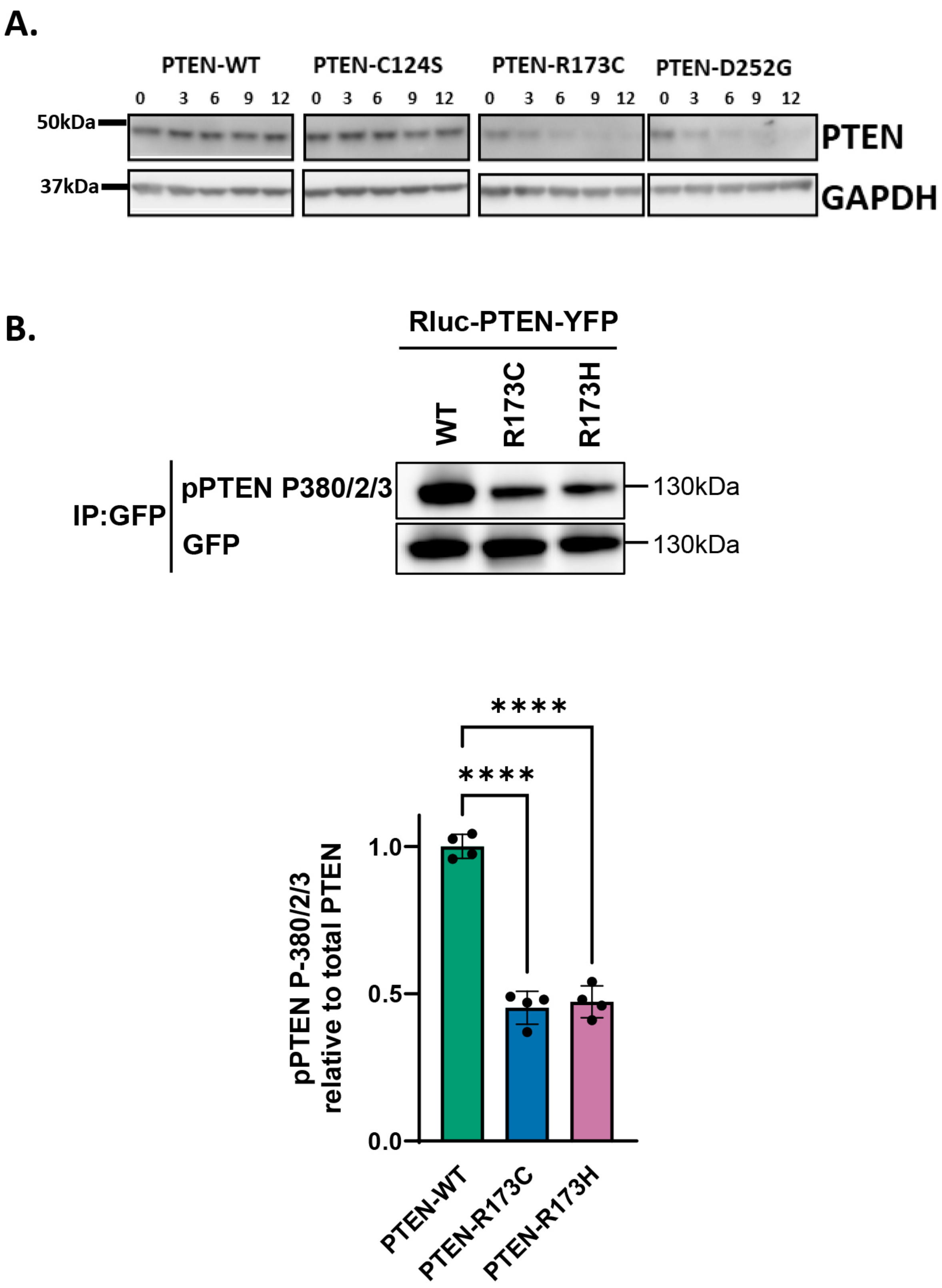
A.
PTEN-WT
PTEN-C124S
PTEN-R173C
PTEN-D252G
0 3 6 9 12
50kDa
37kDa
PTEN
GAPDH
B.
Rluc-PTEN-YFP
WT
R173C
R173H
IP:GFP
pPTEN P380/2/3
GFP
130kDa
130kDa
****
****
pPTEN P-380/2/3 relative to total PTEN
1.0
0.5
0.0
PTEN-WT
PTEN-R173C
PTEN-R173H

Fig. S3. Characterisation of PTEN-R173C. (**A**) Transient lentiviral expression of PTEN wild-type (WT) or the indicated PTEN mutants in U87 cells. The cells were treated with cycloheximide, and PTEN protein expression levels were determined by immunoblotting at the indicated time points. Representative blots are shown. (**B**) Lysates of HEK-293T cells expressing Rluc-PTEN-WT-YFP, Rluc-PTEN-R173C-YFP or Rluc-PTEN-R173H-YFP were immunoprecipitated using anti-GFP antibodies and immunoblotted for PTEN-P-S380/T382/T383. Representative immunoblots from n=4 showing phosphorylated and total PTEN levels. Graph shows quantification of 4 experiments shown as mean±SEM. Statistical analysis was performed using one-way ANOVA. p-values: ****$p<0.0001$.

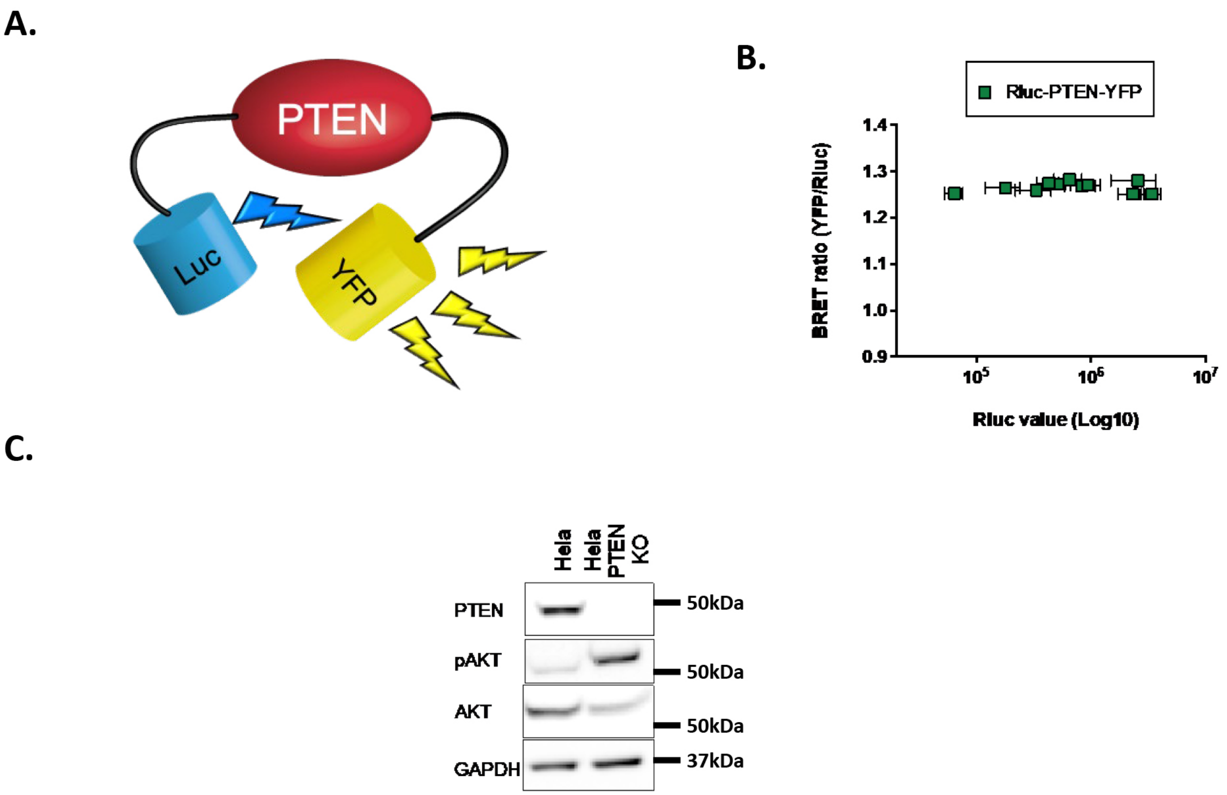

Fig. S4. BRET analysis of PTEN. (**A**) Cartoon showing the intramolecular bioluminescent resonance energy transfer (BRET)-based biosensor (Rluc-PTEN-YFP). The biosensor can reveal dynamic changes in PTEN conformational rearrangement and function, due to shifts in energy transfer between the donor/acceptor couple. (**B**) The conformational readout (measured as BRET ratio) is independent from expression levels of the biosensor, as seen by a stable BRET signal when the biosensor (Rluc-PTEN-YFP) is expressed over 2 log differences (measured as Rluc value). (**C**) Validation of PTEN knock out by immunoblotting in the PTEN-KO HeLa cell line.

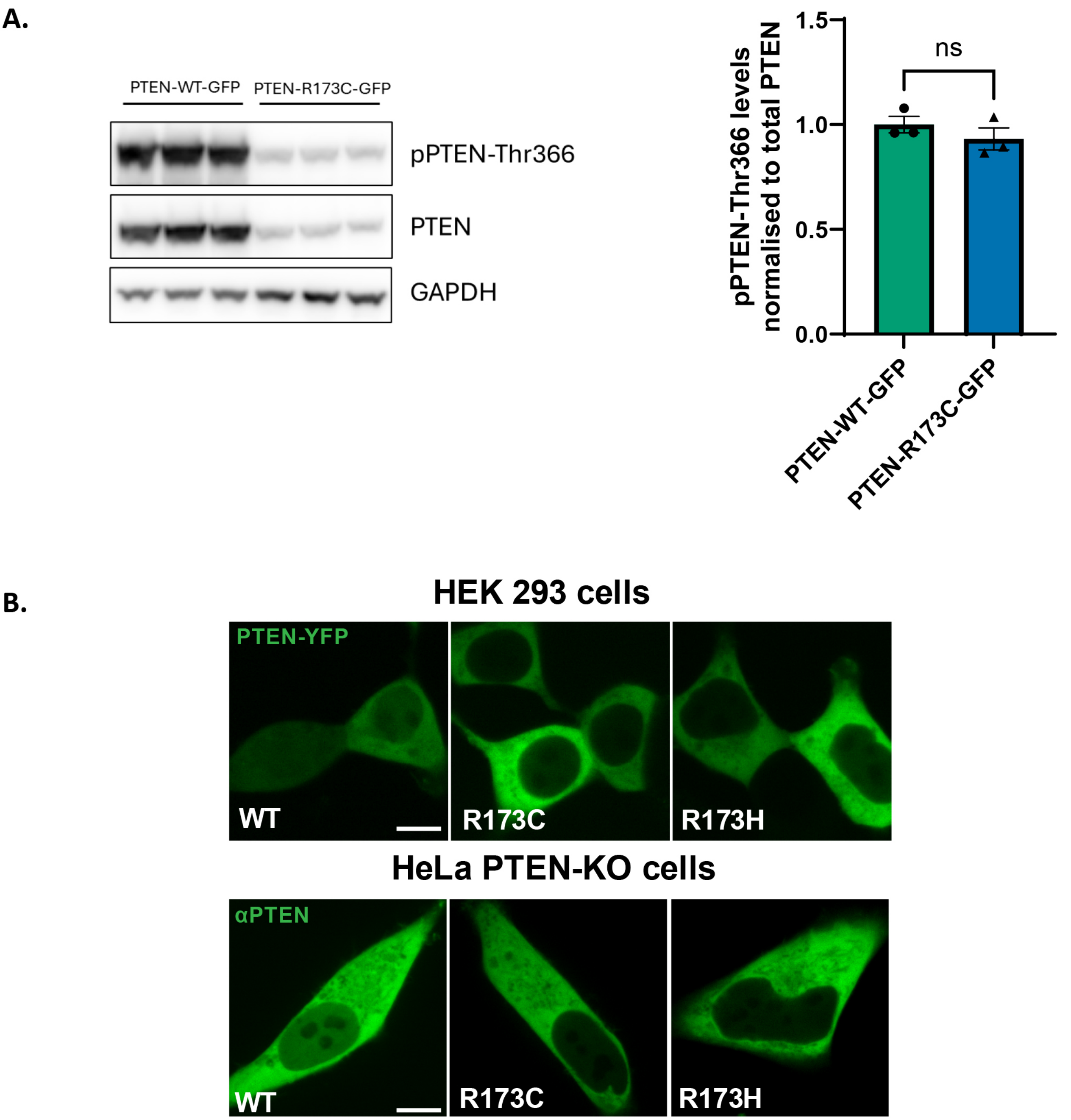

Fig. S5. Investigating protein phosphatase activity and sub-cellular distribution of PTEN-R173 mutants. (**A**) Lenti-X 293T cells were transfected with plasmids expressing PTEN-WT-GFP or PTEN-R173C-GFP. The cells were lysed, and protein extracts were immunoblotted for pPTEN-Thr366 or total PTEN. Graph on right ™shows mean±SEM pPTEN-Thr366 levels normalised to total PTEN levels from n=3 experiments. Statistical analysis was performed using two-tailed unpaired t-test. p-values : ns, not-significant (p>0.05). Corresponding immunoblots shown on the left. (**B**)Live HEK-293T cells or PTEN-KO HeLa cells expressing Rluc-PTEN-WT-YFP, Rluc-PTEN-R173C-YFP or Rluc-PTEN-R173H-YFP were imaged in 4-well µ slides (IBIDI) by confocal microscopy to assess the subcellular localisation of PTEN-YFP. Figure shows representative images from n=3. Scale bar: 10 µm.

Disease Models & Mechanisms • Supplementary information

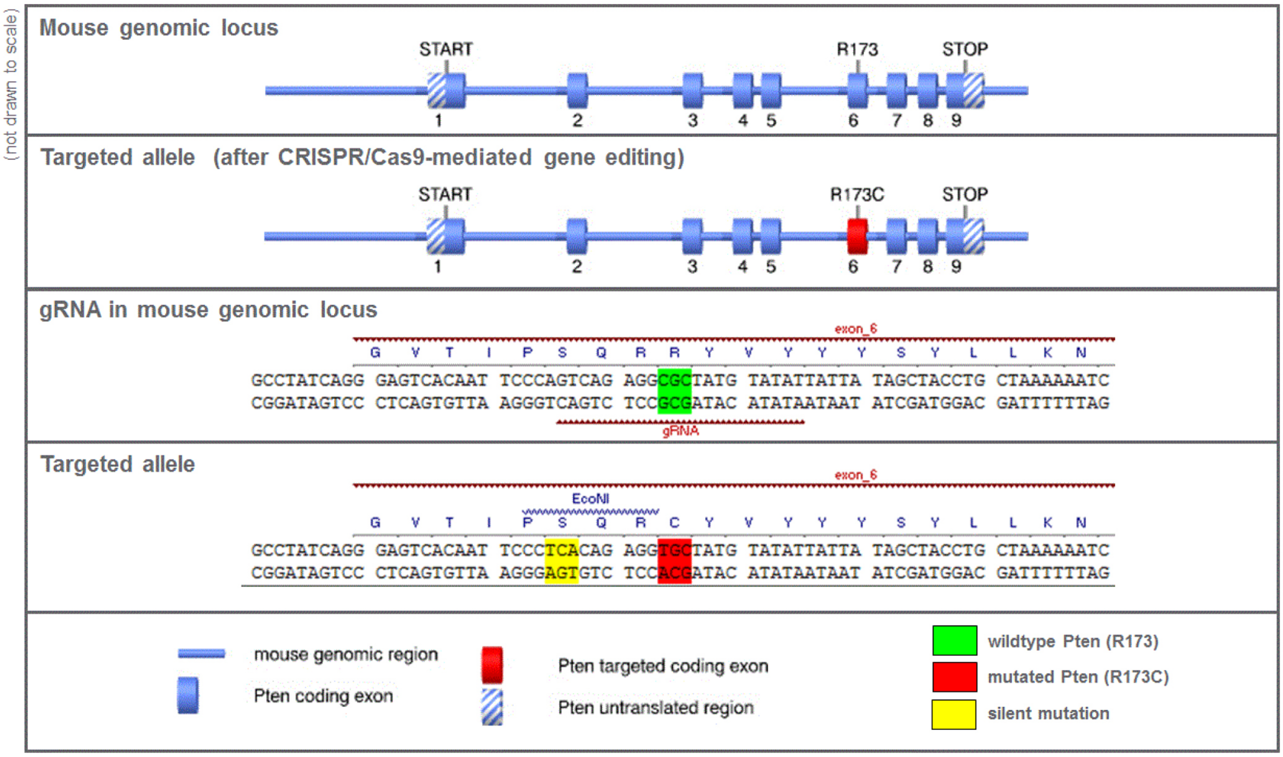

Fig. S6. Generation and characterization of *Pten*$^{+/R173C}$ mice. The R173C mutation was introduced into the mouse *Pten* locus using a CRISPR-Cas9 approach. gRNA was designed to contain the R173C mutation (c.517C>T) and a silent mutation containing the *Eco*NI restriction site upstream of the R173 site, to allow discrimination of wild-type and knock-in allele by PCR analysis. The figure above shows the gRNA sequence and the targeting strategy. gRNA was microinjected into C57BL/6J mouse zygotes along with recombinant Cas9. The zygotes were then transferred to pseudo-pregnant mothers. The litters born (founder animals) were sequenced to check for the incorporation of the R173C mutation and percentage of mosaicism. They were then used as a breeder to generate F1 animals. F1 males positive for the R173C mutation and negative for any off-target mutations were used to generate further animals by IVF using C57BL/6J females.

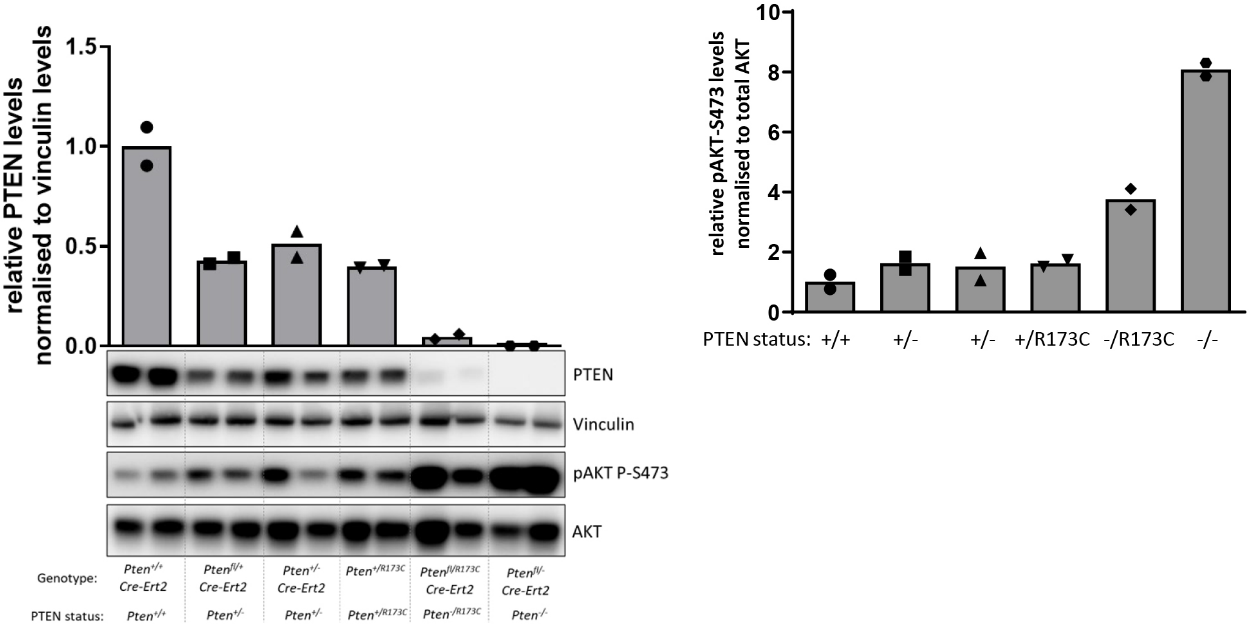

Fig. S7. *In vivo* characterisation of PTEN-R173C. (A) Immunoblots of PTEN protein (quantification in graph on the left) and pAKT-473 (quantification in graph on the right) from MEFs of the indicated genotypes. Quantification shown as mean from 2 MEF lines for each genotype. The MEFs were generated form mice on a C57BL/6J background.

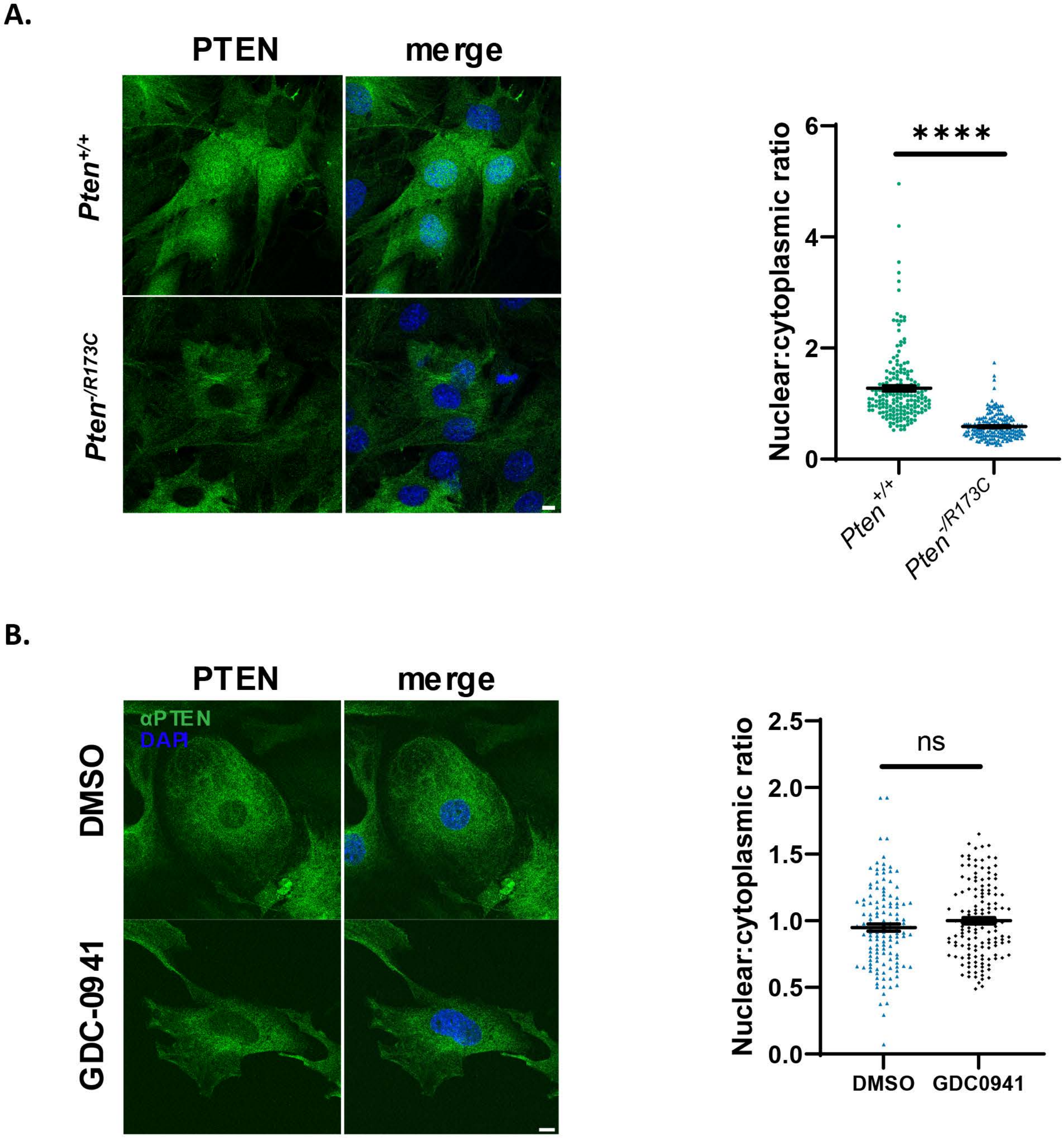

Fig. S8. Sub-cellular localisation of PTEN-R173C. Subcellular localisation of PTEN-R173C. (**A-B**) MEFs of the indicated genotype were immunostained with antibodies to PTEN (green) and DAPI (blue) and imaged by confocal microscopy. Scale bar: 10 µm. Cells in (A) were arrested in the G1/S phase of the cell cycle by double-thymidine block and analysed 6 h after release from thymidine block. Cells in (B) were pre-treated with or without GDC-0941 (1 µM) for 1 h, as indicated. Graphs show nuclear:cytoplasmic ratio of the GFP MFI (mean±SEM from 3 independent experiments). Statistical analysis was performed using two-tailed unpaired t-test. p-values : ****p<0.0001 ; ns, not-significant (p>0.05). The MEFs were generated from mice on a C57BL/6J background.

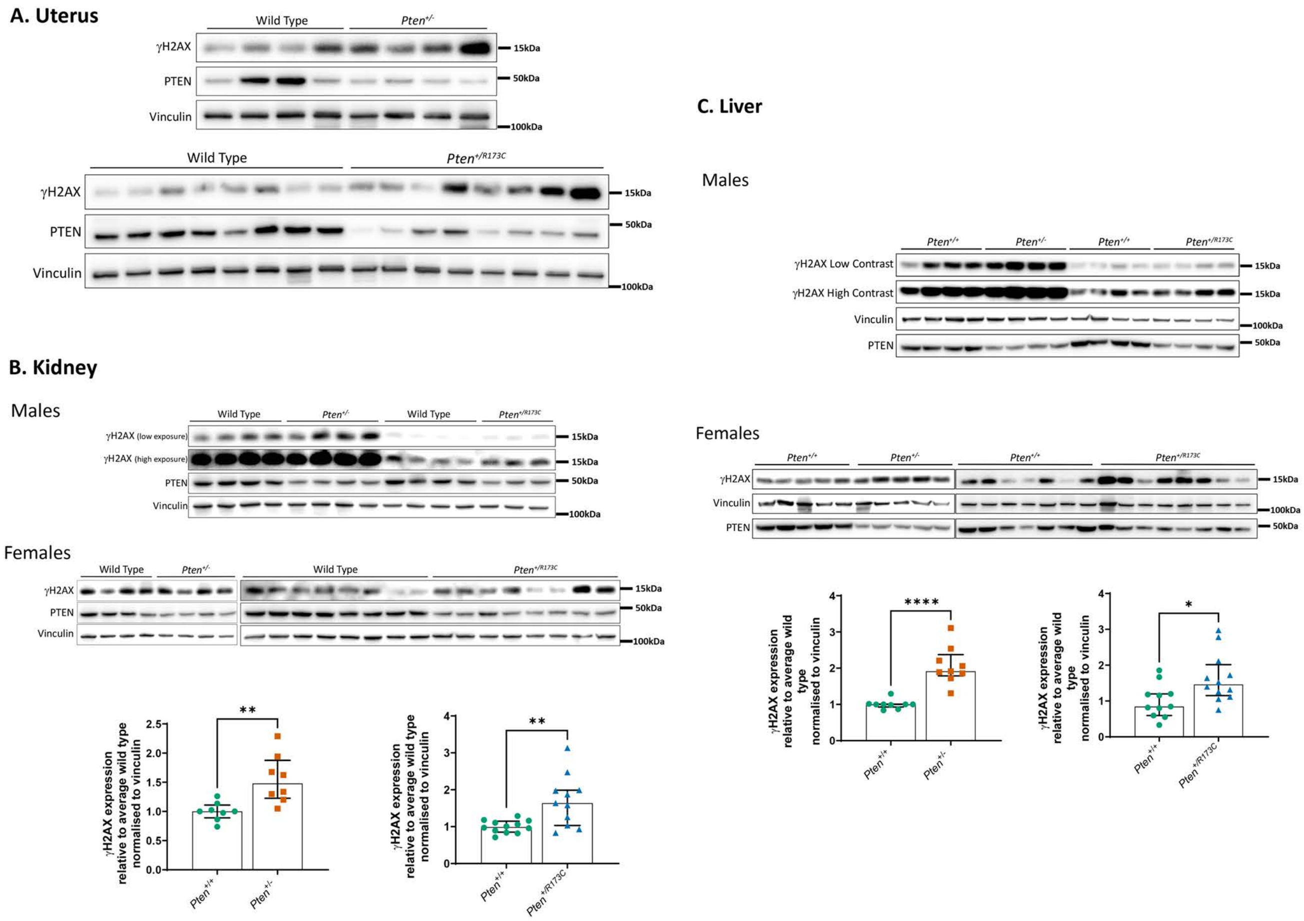

Fig. S9. *In vivo* characterisation of PTEN-R173C. 6-week-old mice on a C57BL/6J background of the indicated genotypes were treated with 7Gy γ-radiation and protein extracts from tissues indicated were used for immunoblotting. Immunoblots showing levels of γH2AX in (**A**) uterus (quantification in Fig. 2G), (**B**) kidney and (**C**) liver. Graphs show quantification of immunoblots. Bars show median with interquartile range. Pairwise comparisons were performed using Mann–Whitney U test. p-values: *p< 0.05; **p<0.01; ****p<0.0001.

A. ***Males***

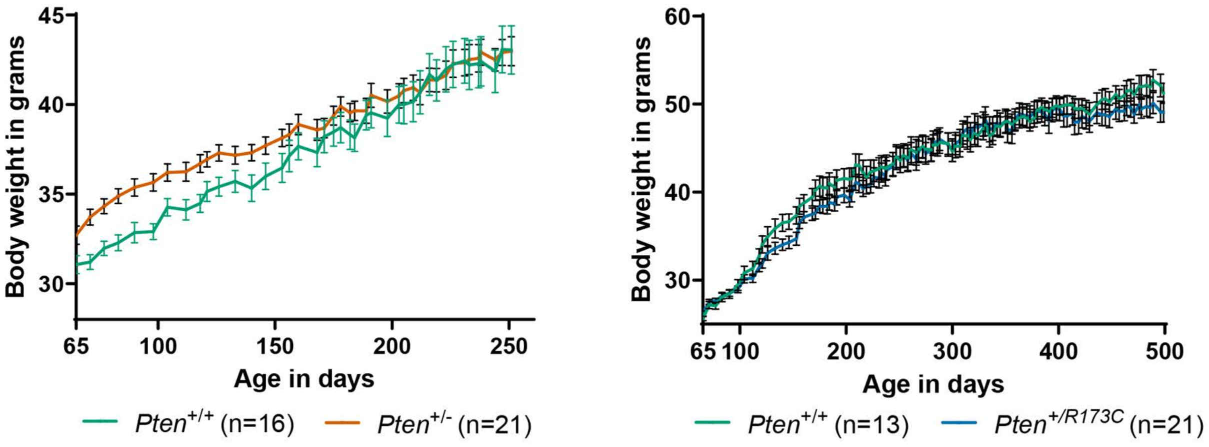

B. ***Females***

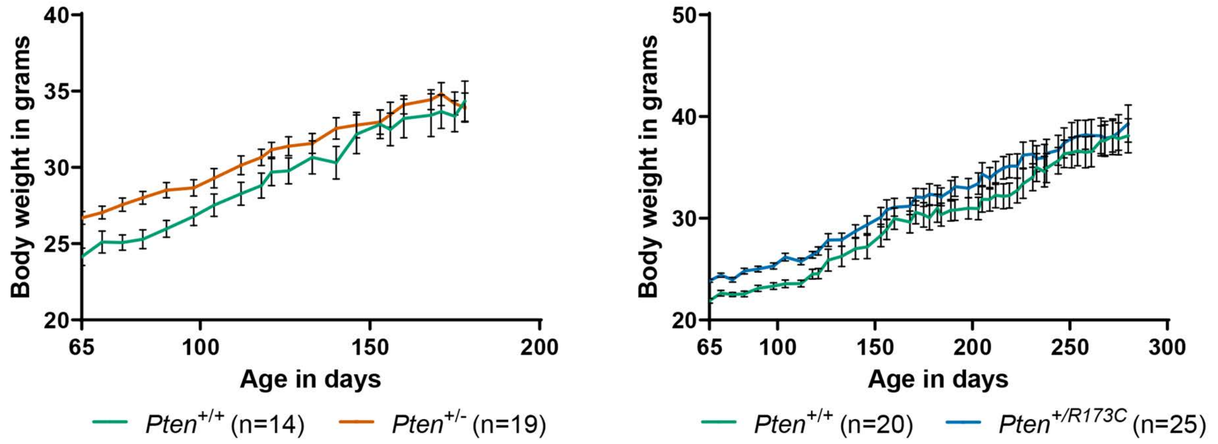

Fig. S10. Age-dependent body weights of *Pten* mutant mice. *Pten*$^{+/R173C}$ and *Pten*$^{+/-}$ mice, and their littermate controls, on a mixed C57BL/6J x Sv/129 background were allowed to age. The mice weighed once a week. Data show mean±SEM.

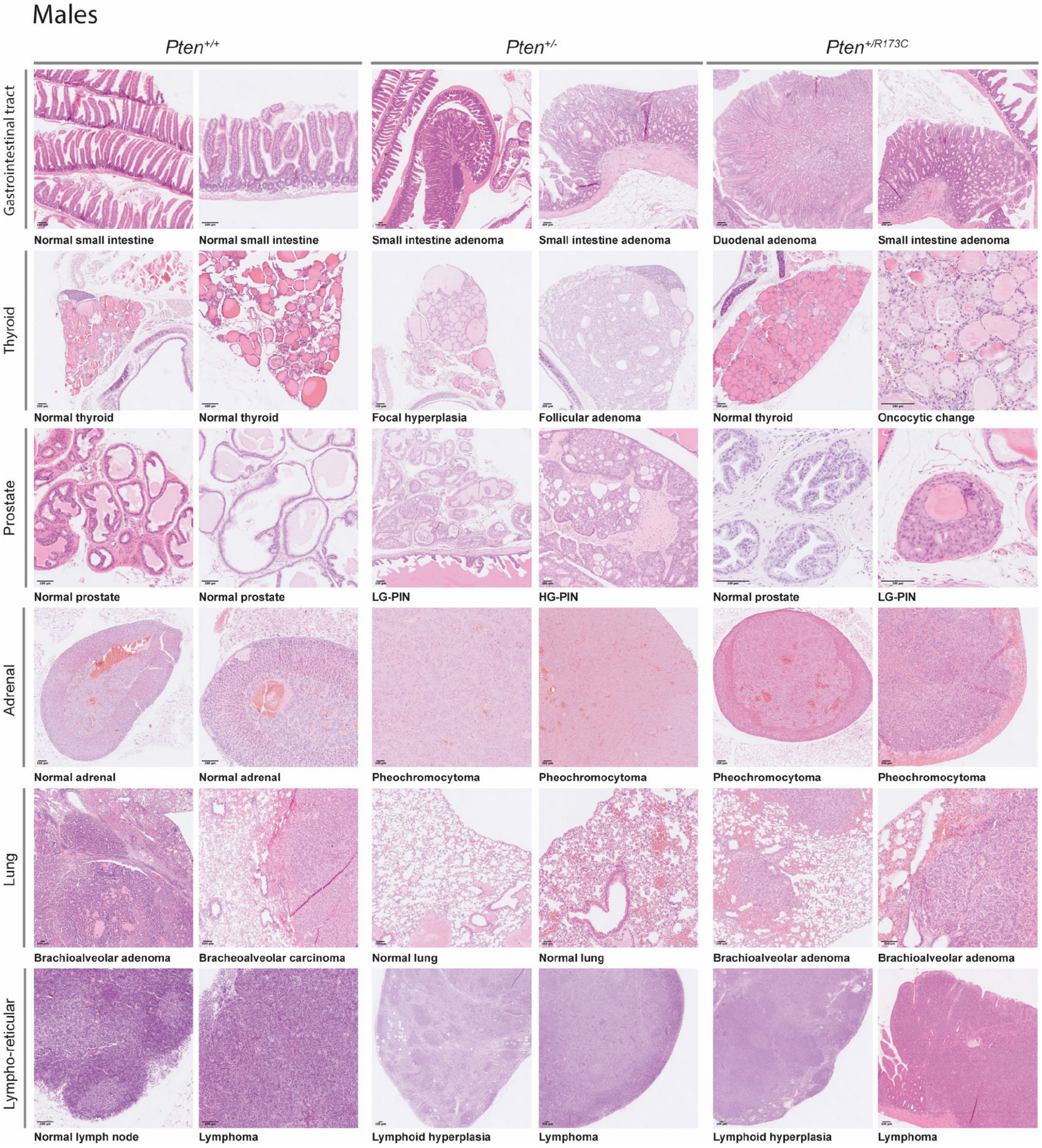

Fig. S11. Histopathological analysis of male *Pten*$^{+/R173C}$ mice. *Pten*$^{+/R173C}$ and *Pten*$^{+/-}$ mice, and their littermate control *Pten*$^{+/+}$ mice, on a mixed C57BL/6J x Sv129 background were allowed to age. Mice were euthanised for welfare reasons (ill health or masses with a combined size of ≥ 1.4 cm^2 surface area) or at a specified age. Representative photomicrographs of H&E-stained sections of indicated tissues from male mice. Scale bar 100µm

Disease Models & Mechanisms • Supplementary information

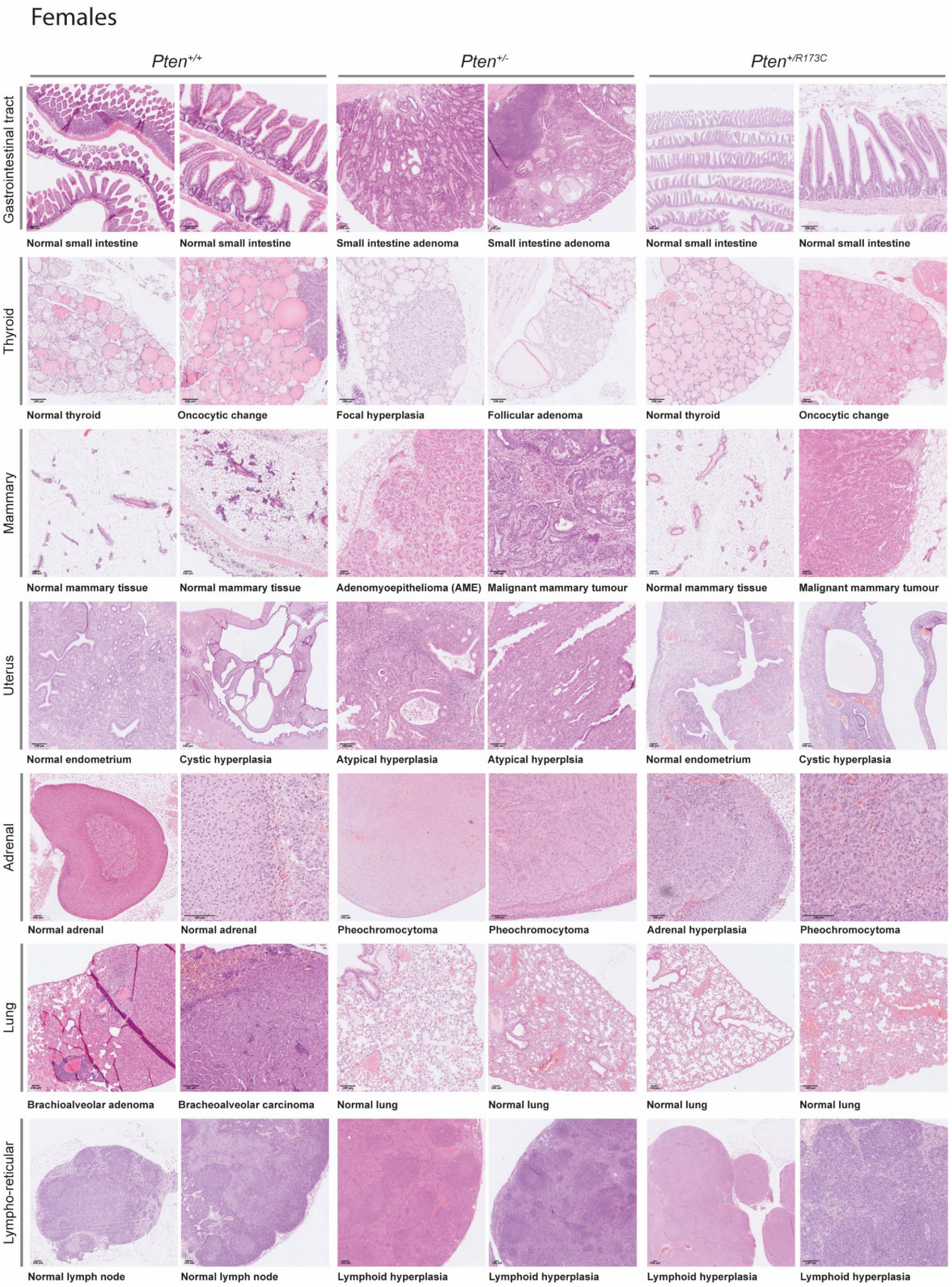

Fig. S12. Histopathological analysis of female *Pten*$^{+/R173C}$ mice. *Pten*$^{+/R173C}$ and *Pten*$^{+/-}$ mice, and their littermate control *Pten*$^{+/+}$ mice, on a mixed C57BL/6J x Sv129 background were allowed to age. Mice were euthanised for welfare reasons (ill health or masses with a combined size of ≥ 1.4 cm^2 surface area) or at a specified age. Representative photomicrographs of H&E-stained sections of indicated tissues from female mice. Scale bar 100µm.

Disease Models & Mechanisms • Supplementary information

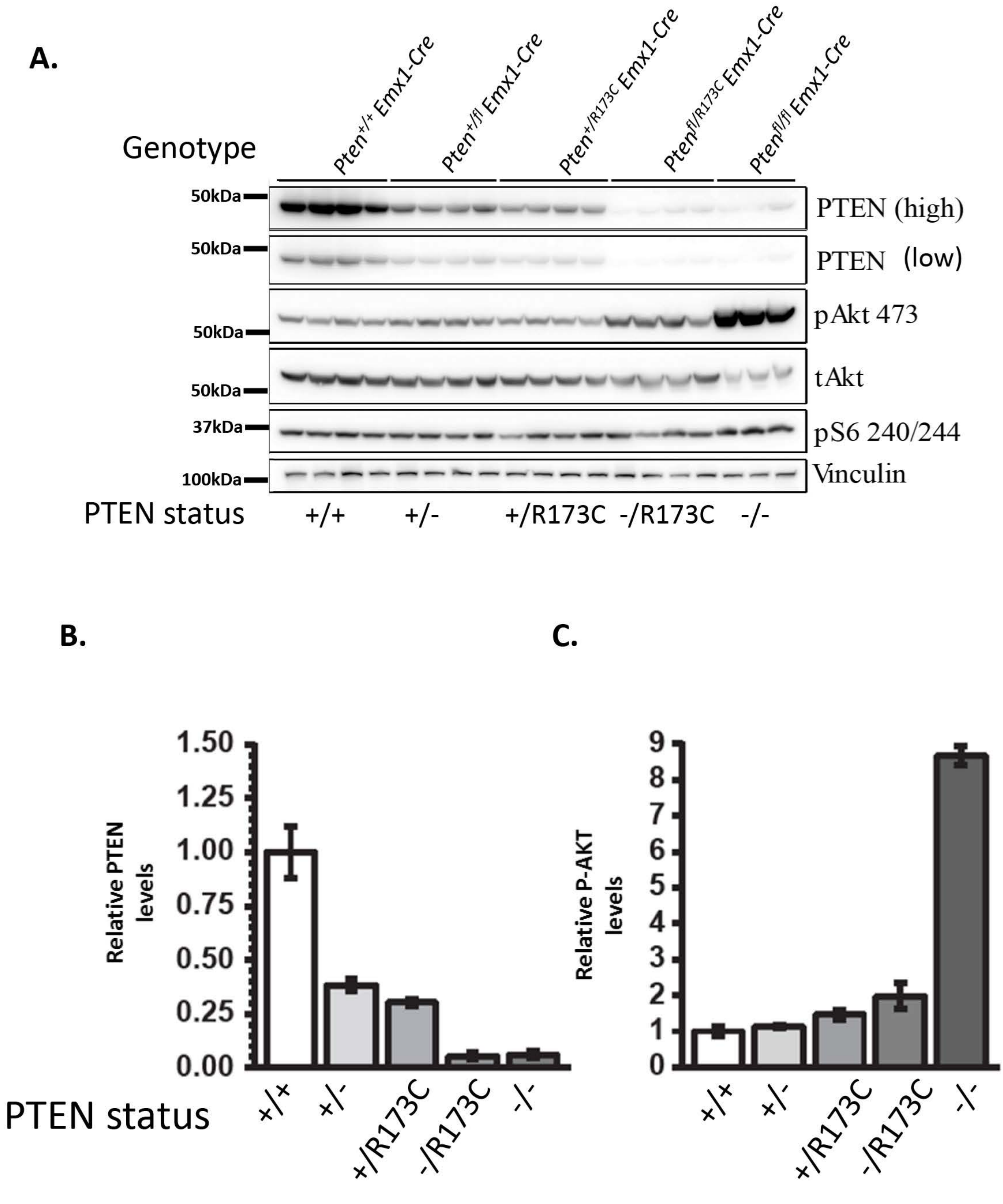

Fig. S13. Expression of PTEN and downstream pathway component in brain cortical lysates. (**A**) Immunoblots of PTEN protein and downstream PI3K pathway components in cortico-hippocampal lysates of postnatal pups (P1/P2) from the indicated genotypes. (**B**) Quantification of PTEN protein, normalised to the vinculin loading control. Data show mean±SEM (**C**) Quantification of p-AKT normalised to total AKT. n=4 for all mutants except for *Pten*$^{fl/fl}$;*Emx1-Cre* where n=3. Data show mean±SEM. All mice were on a C57BL/6J background

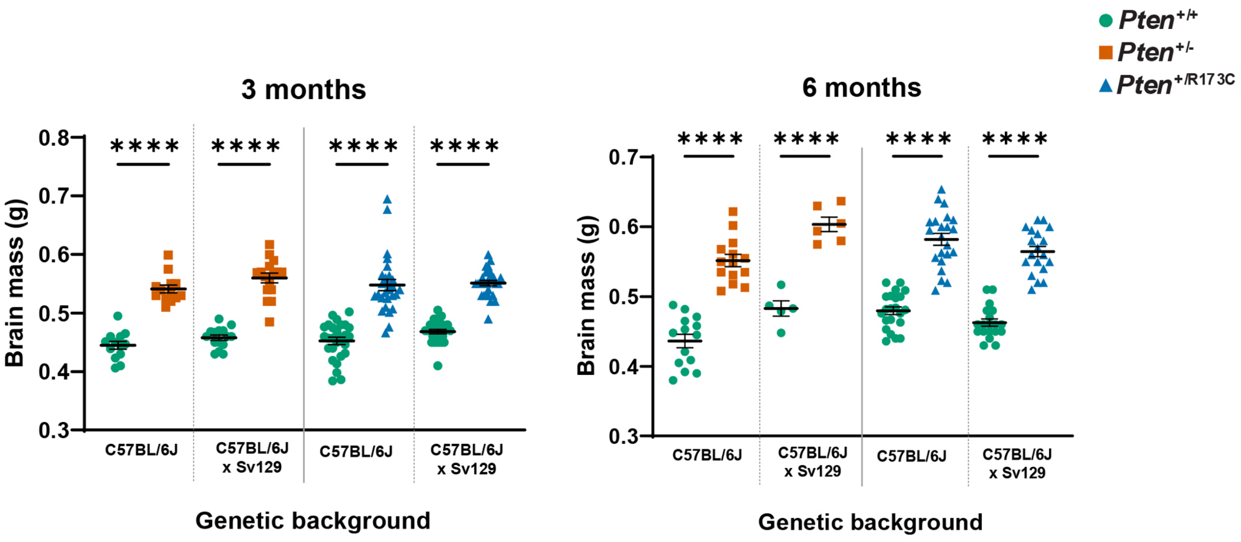

Fig. S14. Analysis of brain mass in mice. Brain mass of *Pten*$^{+/-}$, *Pten*$^{+/R173C}$ and *Pten*$^{+/+}$ littermate controls of both sexes at 3 and 6 months of age on the indicated genetic backgrounds. Data show mean ± SEM, statistical analysis done using One-way ANOVA followed by Tukey's multiple comparison test, ****$p<0.0001$

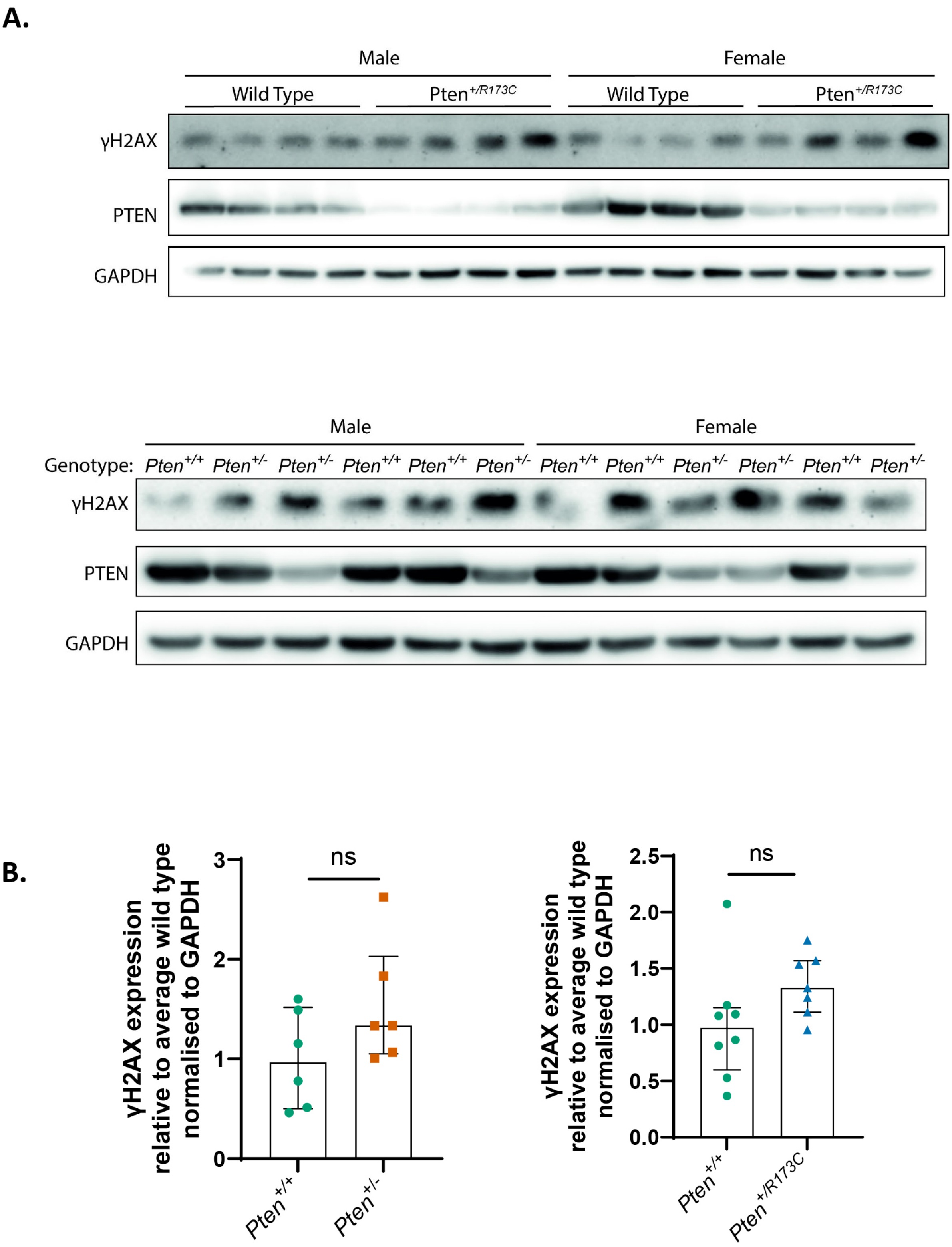

Fig. S15. *In vivo* characterisation of PTEN-R173C. (**A**) 6-week-old mice on a C57BL/6J background of the indicated genotypes were treated with 7Gy γ-radiation and protein extracts from brain were used for immunoblotting. Immunoblots showing levels of γH2AX. (**B**) Graphs show quantification of immunoblots. Bars show median with interquartile range. Pairwise comparisons were performed using Mann–Whitney U test. ns, not-significant ($p > 0.05$).

Disease Models & Mechanisms • Supplementary information

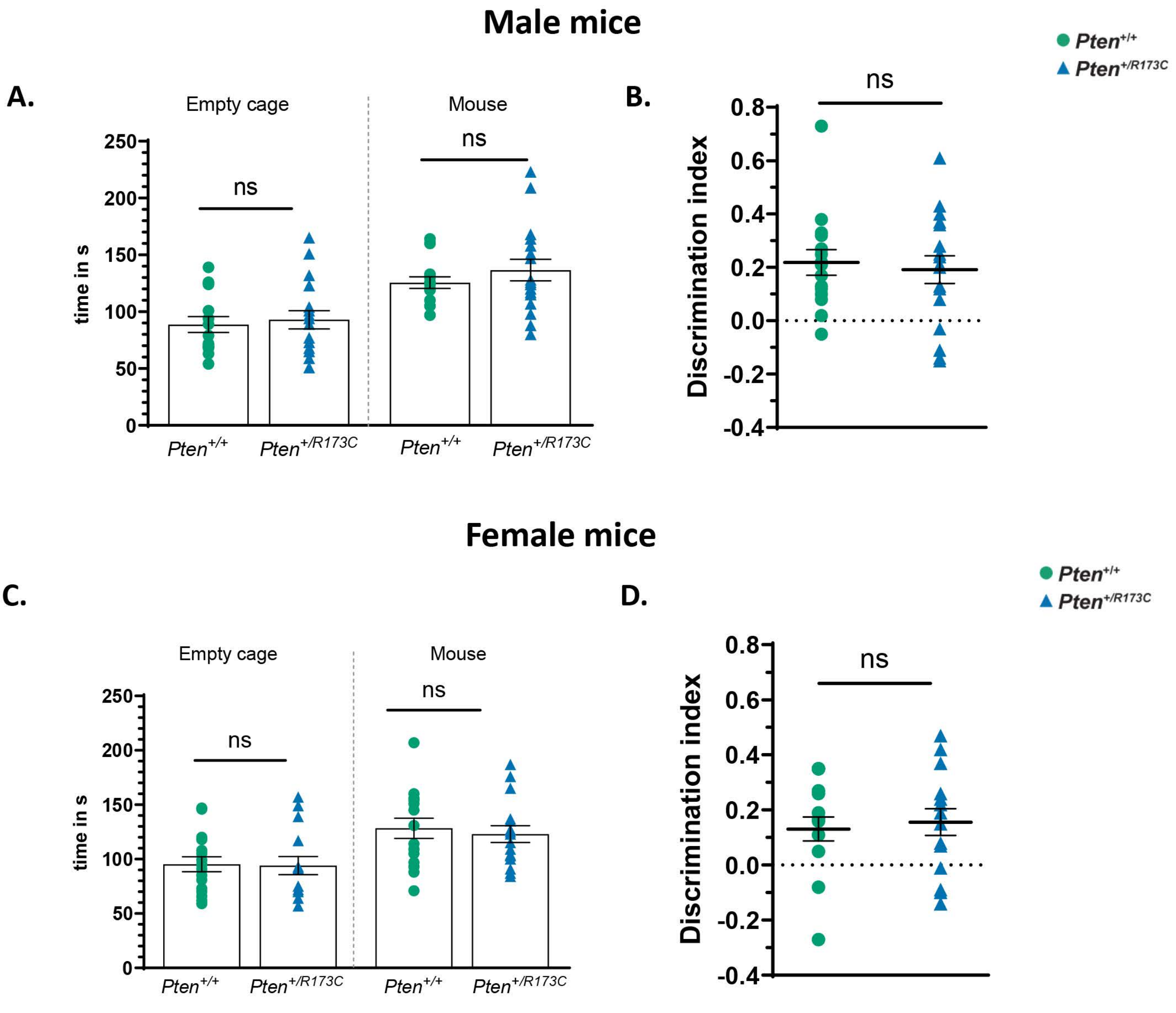

Fig. S16. Lack of sociability deficits in *Pten*$^{+/R173C}$ mice in Crawley's sociability test. Analysis of sociability of male (A) and female (C) *Pten*$^{+/R173C}$ mice and littermate controls. In Crawley's three chamber test, mice were analysed for their preference of a mouse versus an empty cage. Data show mean±SEM analysed using Ordinary one-way ANOVA. (B and D) Discrimination indexes for male and female WT and *Pten* mutant animals analysed in A and C. Males: n=14 for *Pten*$^{+/+}$ and n=17 for *Pten*$^{+/R173C}$ mice. Females: n=16 mice of each genotype. Data show mean SEM, statistical analysis done using Unpaired Student's t-test. ns, not-significant (p>0.05).

Disease Models & Mechanisms • Supplementary information

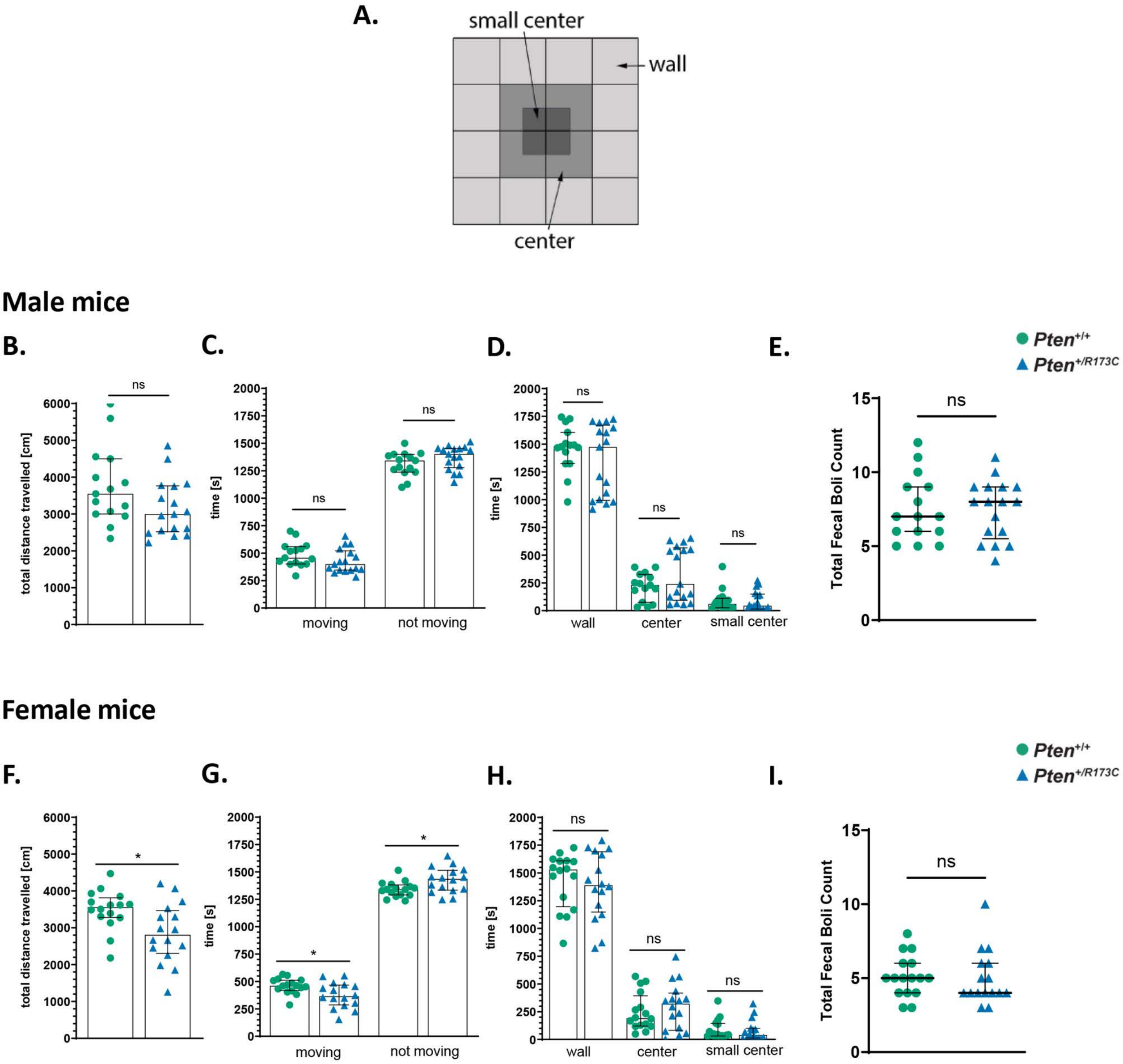

Fig. S17. Female *Pten*$^{+/R173C}$ mice show reduction in exploratory locomotion in the open field test. (**A**). Scheme of the open field arena. Parameters measured in the open field test for male (**B, C, D and E**), and female (**F, G, H, I**) *Pten*$^{+/R173C}$ mice and their littermate WT controls. Total distance travelled (**B and F**), time spent moving and not moving (**C and G**), time spent in each of the three zones as indicate in A (**D and H**) and defecation during the test (**E and I**). There were no differences between *Pten*$^{+/R173C}$ and control male mice. Female *Pten*$^{+/R173C}$ mice spent less time moving and travel smaller distances compared to their WT littermate controls indicating possible exploratory locomotion deficits. Males: n=15 for WT and n=17 *Pten*$^{+/R173C}$ mice. Females: n=16 mice of each genotype. Data show median with interquartile range. Two-tailed Mann-Whitney U test. p-values : *$p < 0.05$; ns, not-significant ($p > 0.05$).

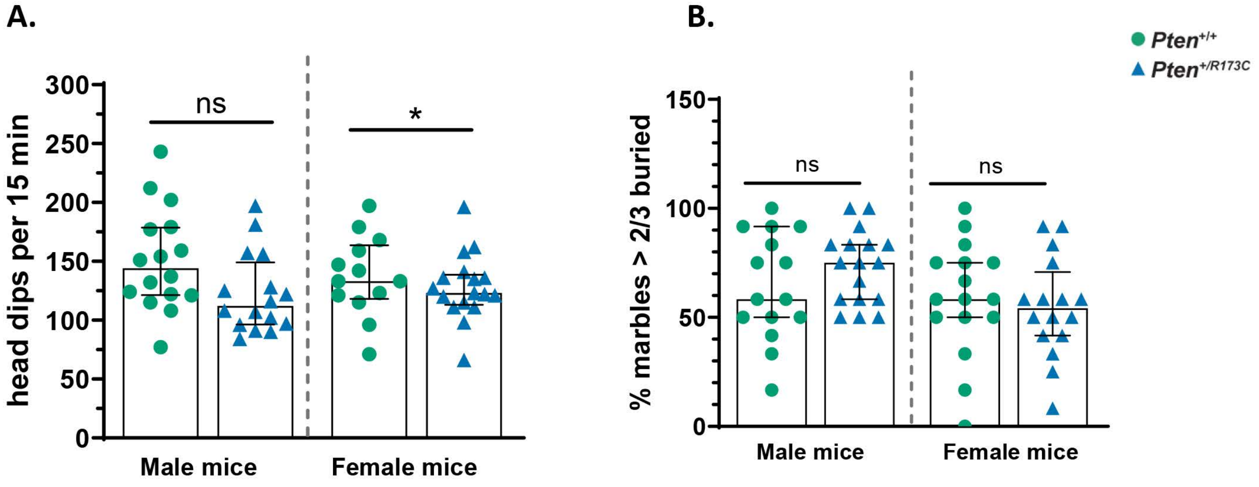

Fig. S18. Female *Pten*$^{+/R173C}$ mice show reduction in exploratory locomotion in the hole board test. (**A**)**.** Hole board test: female *Pten*$^{+/R173C}$ mice made significantly fewer head dips than *Pten*$^{+/+}$ littermate controls. Males: n=13 *Pten*$^{+/+}$ and 17 *Pten*$^{+/R173C}$ mice. Females n=16 mice per genotype. Data show mean ± SEM. Two-tailed Mann-Whitney U test. *p<0.05. (**B**)**.** Marble Burying test: there was no significant difference between *Pten*$^{+/R173C}$ and *Pten*$^{+/+}$ controls of either sex in this test. Males: n=15 for *Pten*$^{+/+}$ and n=17 for *Pten*$^{+/R173C}$ mice. Females n=16 mice per genotype. Data show median with interquartile range. Two-tailed Mann-Whitney U test. p-values : *p< 0.05; ns, not-significant (p>0.05).

Table S1. Clinical findings of PHTS patients with nuclear excluded but catalytically active PTEN variants

Available for download at
https://journals.biologists.com/dmm/article-lookup/doi/10.1242/dmm.052527#supplementary-data

Table S2. Clinical findings of PHTS patients with PTEN-R173 mutations

Available for download at
https://journals.biologists.com/dmm/article-lookup/doi/10.1242/dmm.052527#supplementary-data

Table S3. Reason for euthanasia of $Pten^{+/+}$, $Pten^{+/-}$ and $Pten^{+/R173C}$ mice

Available for download at
https://journals.biologists.com/dmm/article-lookup/doi/10.1242/dmm.052527#supplementary-data

Table S4. Frequency and grade of lesions in male mice

Available for download at
https://journals.biologists.com/dmm/article-lookup/doi/10.1242/dmm.052527#supplementary-data

Table S5. Frequency and grade of lesions in female mice

Available for download at
https://journals.biologists.com/dmm/article-lookup/doi/10.1242/dmm.052527#supplementary-data

Table S6. Frequency and grade of genetic background specific and immune findings in male mice

Available for download at
https://journals.biologists.com/dmm/article-lookup/doi/10.1242/dmm.052527#supplementary-data

Table S7. Frequency and grade of immune findings in female mice

Available for download at
https://journals.biologists.com/dmm/article-lookup/doi/10.1242/dmm.052527#supplementary-data

Table S8. DNA constructs

Available for download at
https://journals.biologists.com/dmm/article-lookup/doi/10.1242/dmm.052527#supplementary-data

Table S9. Primers for site-directed mutagenesis

Available for download at
https://journals.biologists.com/dmm/article-lookup/doi/10.1242/dmm.052527#supplementary-data

Table S10. Primers used for genotyping

Available for download at
https://journals.biologists.com/dmm/article-lookup/doi/10.1242/dmm.052527#supplementary-data

Disease Models & Mechanisms • Supplementary information

Table S11. Antibodies used

Available for download at
https://journals.biologists.com/dmm/article-lookup/doi/10.1242/dmm.052527#supplementary-data

Table S12. qRT PCR primers

Available for download at
https://journals.biologists.com/dmm/article-lookup/doi/10.1242/dmm.052527#supplementary-data

Table S13. Statistical tests and p values

Available for download at
https://journals.biologists.com/dmm/article-lookup/doi/10.1242/dmm.052527#supplementary-data

Disease Models & Mechanisms • Supplementary information
